# *Panax notoginseng*-Derived Carbon Dots Herbzymes Ameliorate Renal Ischemia-Reperfusion Injury via Anti-Inflammation, Antioxidation and Gut-Kidney Axis Regulation

**DOI:** 10.7150/thno.126643

**Published:** 2026-01-14

**Authors:** Mei Yang, Zhenting Zhao, Zhichao Deng, Shirui Sun, Yingcong Guo, Zepeng Li, Bingxuan Zheng, Chengbo Zhang, Jianhui Li, Hongbao Li, Mingzhen Zhang, Hua Wang, Kun Ye, Wujun Xue, Chenguang Ding

**Affiliations:** 1 Department of Organ Procurement and Allocation, the First Affiliated Hospital of Xi'an Jiaotong University, Xi'an, Shaanxi, 710061, China.; 2 Department of Kidney Transplantation, the First Affiliated Hospital of Xi'an Jiaotong University, Xi'an, Shaanxi, 710061, China.; 3 Institute of Organ Transplantation, Xi'an Jiaotong University, Xi'an, Shaanxi, 710061, China.; 4 School of Basic Medical Sciences, Xi'an Jiaotong University, Xi'an, Shaanxi, 710061, China.; 5 Department of Hepatobiliary and Pancreatic Surgery, Key Laboratory of Artificial Organs and Computational Medicine in Zhejiang Province, Shulan (Hangzhou) Hospital, Hangzhou, Zhejiang, 310022, China.; 6 Department of Nephrology, Guangxi Clinical Research Center for Chronic Kidney Disease, People's Hospital of Guangxi Zhuang Autonomous Region, Nanning, Guangxi, 530021, China.

**Keywords:** renal ischemia-reperfusion injury, herbzyme, *Panax notoginseng*, gut-kidney axis, tryptophan, inflammation, oxidative stress

## Abstract

Renal ischemia-reperfusion injury (RIRI) mainly comes from inflammation and oxidative stress. Treating with inflammation monotherapy or oxidative stress monotherapy can't reduce RIRI. This study involved the synthesis of *Panax notoginseng*-derived carbon dots (PN-CDs) herbzymes, nanozymes from Chinese herbal medicines, exhibiting inherent antioxidant enzymatic activity. The diverse surface functional groups facilitate the effective scavenging of reactive oxygen species (ROS) and suppress the expression of inflammatory factors. In the RIRI model, PN-CDs demonstrated extended systemic circulation and improved renal targeting, effectively decreasing renal tissue concentrations of neutrophil gelatinase-associated lipocalin (NGAL), creatinine, blood urea nitrogen, and kidney injury molecule-1 (KIM-1). They also decreased inflammatory factors and lipid peroxidation markers, thereby mitigating renal damage. Multi-omics analysis showed that PN-CD protects kidney function mainly via gut microbiota, shrinking nephrotoxins such as indoxyl sulfate and enhancing beneficial metabolisms such as β-indol-3-acetamide and stimulating pathways like aryl hydrocarbon receptor (AHR), ERK to reduce inflammation and oxidative stress. In conclusion, PN-CDs herbzyme was a potential treatment for RIRI and provided the theoretical basis for the herbzyme therapy of kidney disease, as well as the proof-of-concept for the gut-kidney axis.

## 1. Introduction

Renal ischemia-reperfusion injury (RIRI) involves the deterioration of kidney tissue damage that happens when the blood supply is resumed after ischemia^1^. In a clinical setting, RIRI is commonly seen in situations like cardiac surgery, kidney transplants, hypovolemic shock, very low blood pressure, a sudden blockage in the blood flow to the kidneys (acute renal artery occlusion), and serious infections (sepsis)^2^. RIRI stands for the inevitable renal insult of organ procurement, which can lead to acute renal failure or delayed graft function^3^. The underlying molecular mechanisms for RIRI have yet to be clearly understood; however, processes such as inflammation, oxidative stress, apoptosis, ferroptosis, and so on are involved^4^.

Oxidative stress and inflammation are very important in the development and progression of RIRI. Excessive reactive oxygen species (ROS) can result in problems with mitochondrial function, lipid peroxidation, ferroptosis, and various types of harm to the epithelial cells of the renal tubules^5^. This cellular damage triggers an inflammatory response that further exacerbates kidney injury: inflammatory cells are recruited to the site, forming a self-amplifying inflammatory loop^6^, ^7^. Excessive ROS during renal reperfusion compromises endogenous antioxidant enzyme activity, causing structural damage to the cytoskeleton and other cellular components^8^. Therefore, scavenging ROS and anti-inflammation are effective strategies for treating RIRI. Although various nano-antioxidants have been developed, such as multifunctional nanoplatforms based on Fe₃O₄ and strontium ions^9^, ultrasmall nanomicelles^10^, and cerium dioxide nanoparticles^11^, the complexity of synthesis and safety concerns of these nano-formulations have hindered their clinical translation. Therefore, it is essential to create environmentally friendly, safe, and economical antioxidants for RIRI treatment.

Beyond oxidative stress and inflammation, recent researches have shown that gut microbiota and their metabolites are crucial in the development and advancement of RIRI^12^, ^13^. On one hand, dysregulated gut microbiota produce renal toxins, which directly enter the bloodstream and exacerbate kidney injury^14^. Two of the most recognized uremic toxins produced in the gut are indoxyl sulfate and p-cresyl sulfate. Research indicates that gut microbiota break down tryptophan in the intestine to generate indole, which is subsequently absorbed and metabolized in the liver to produce indoxyl sulfate^15^. Indoxyl sulfate can activate inflammatory signaling pathways, induce oxidative stress, promote mitochondrial dysfunction and cell apoptosis, thereby exacerbating RIRI^16^. Conversely, gut microbiota can modulate both local and systemic immune responses, potentially causing excessive immune activation and exacerbating renal inflammation and oxidative stress^17^, ^18^. Therefore, regulating gut microbiota-tryptophan metabolism may also be a potential strategy and mechanism for alleviating RIRI.

Carbon dots (CDs) are zero-dimensional carbon nanomaterials, generally less than 10 nm in size, renowned for their fluorescent emission properties^19^. CDs possess a sp²-conjugated carbon core, and various surface functional groups endow them with distinct optical properties and unique pharmacological activities^20^. Owing to their outstanding photoluminescent properties, photostability, water solubility, biocompatibility, and ease of chemical modification, carbon nanotubes have found extensive application in fields such as fluorescent labelling, bioimaging, drug delivery, disease treatment, and biosensing^21^. However, traditional CDs are usually carbonized polymer dots with an organic-inorganic hybrid structure, exhibiting poor biocompatibility along with risks of slow metabolism in the body and potential accumulation. In addition, they are typically synthesized via a bottom-up strategy, which requires surface modification or the use of toxic chemical reagents, resulting in complex processes and high environmental risks. More and more people pay attention to green chemistry and CD isolation and utilization from all kinds of green natural chemical ingredients, such as Chinese medicine, vegetables, fruits, etc., have been intensively studied^22^. We proposed the concept of "herbzyme" in 2025, referring to nanozymes with diverse enzymatic functions derived from Chinese herbal medicines through processing, chelation, self-assembly, or other methods^23^. Herbzymes are classified into three primary groups according to their synthesis methods: herb CD nanozymes, polyphenol-metal nanozymes, and herb extract nanozymes^23^. Herb CD nanozymes not only possess the fluorescent properties of CDs but also demonstrate enzyme-like activities, replicating the catalytic roles found in natural antioxidant systems, including functions like peroxidase (POD), superoxide dismutase (SOD), and catalase (CAT). They exhibit a strong ability to scavenge reactive oxygen and nitrogen species (RONS). Herb CD nanozymes offer superior broad-spectrum ROS scavenging, enhanced stability, rapid clearance, cost-effectiveness, and scalability compared to traditional antioxidants, making them widely applicable in treating oxidative stress and inflammation-related diseases^24^-^26^. As a traditional Chinese medicine, *Panax notoginseng* can alleviate RIRI through multiple pathways such as antioxidation, anti-inflammation, and inhibition of cell apoptosis^27^. Therefore, synthesizing *Panax notoginseng* CD nanozymes (PN-CDs) using *Panax notoginseng* as a precursor holds promising prospects for RIRI treatment.

**Figure 1:**
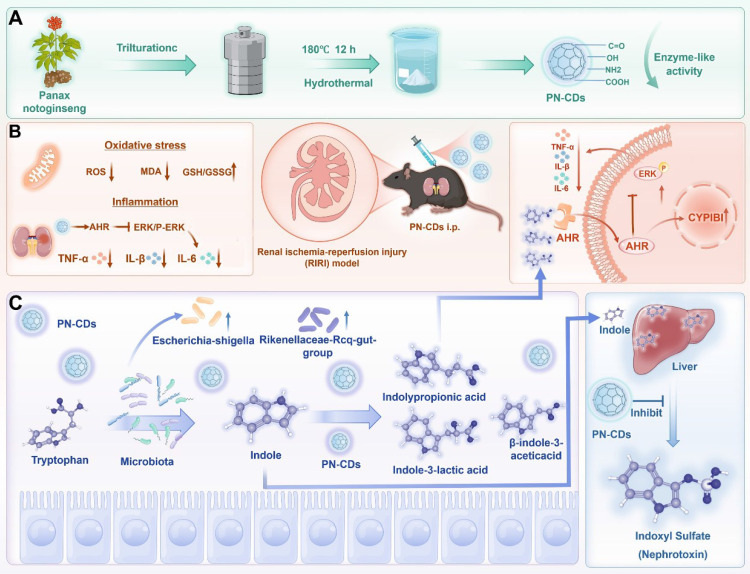
Schematic illustration of PN-CDs synthesis and its mechanism in alleviating RIRI. **A.** PN-CDs were synthesized via a hydrothermal method, with surface-functionalized carboxyl groups, hydroxyl groups, amino groups, and carbonyl groups. These chemical groups endow PN-CDs with enzyme-like activities. **B.** PN-CDs can alleviate oxidative stress and inflammation by activating the AHR and inhibiting ERK signaling pathway. **C.** PN-CDs regulate gut microbiota and tryptophan-indole metabolism by inhibiting the synthesis of renal toxin indoxyl sulfate and promoting the synthesis of indolylpropionic acid, β-indole-3-acetic acid, and indole-3-lactic acid. These metabolites further activate AHR and inhibit the ERK signaling pathway to exert anti-inflammatory effects.

In this study, we selected* Panax notoginseng* as a precursor to synthesize herb CDs, which were characterized to possess favorable antioxidant enzyme-like activities, and thus we termed them "herbzymes". Research conducted showed that PN-CDs herbzymes significantly reduced excessive ROS production and inflammation in lipopolysaccharide (LPS)-induced RAW 264.7 cell and HK-2 cell hypoxia-reoxygenation (H/R) models. In a mouse model of RIRI, PN-CDs significantly alleviated renal tubular injury and oxidative stress responses. Multi-omics analysis revealed that PN-CDs influence the gut microbiota in RIRI model mice, adjust tryptophan-indole metabolites, decrease renal toxin indoxyl sulfate, increase levels of metabolites such as β-indole-3-acetic acid (IAA), indolylpropionic acid (IPA), and indole-3-lactic acid (ILA), trigger the aryl hydrocarbon receptor (AHR) signaling pathway, and reduce the ERK inflammatory signaling pathway. In summary, PN-CDs can alleviate RIRI through multiple targets, including reducing inflammatory responses, inhibiting oxidative stress, and regulating the gut microbiota-tryptophan-AHR metabolism pathway **(Figure 1)**. This study offers a theoretical foundation for using herbzymes in RIRI and further supports the gut-kidney axis theory.

## 2. Results

### 2.1 Fabrication and analysis of PN-CDs

**Figure 2:**
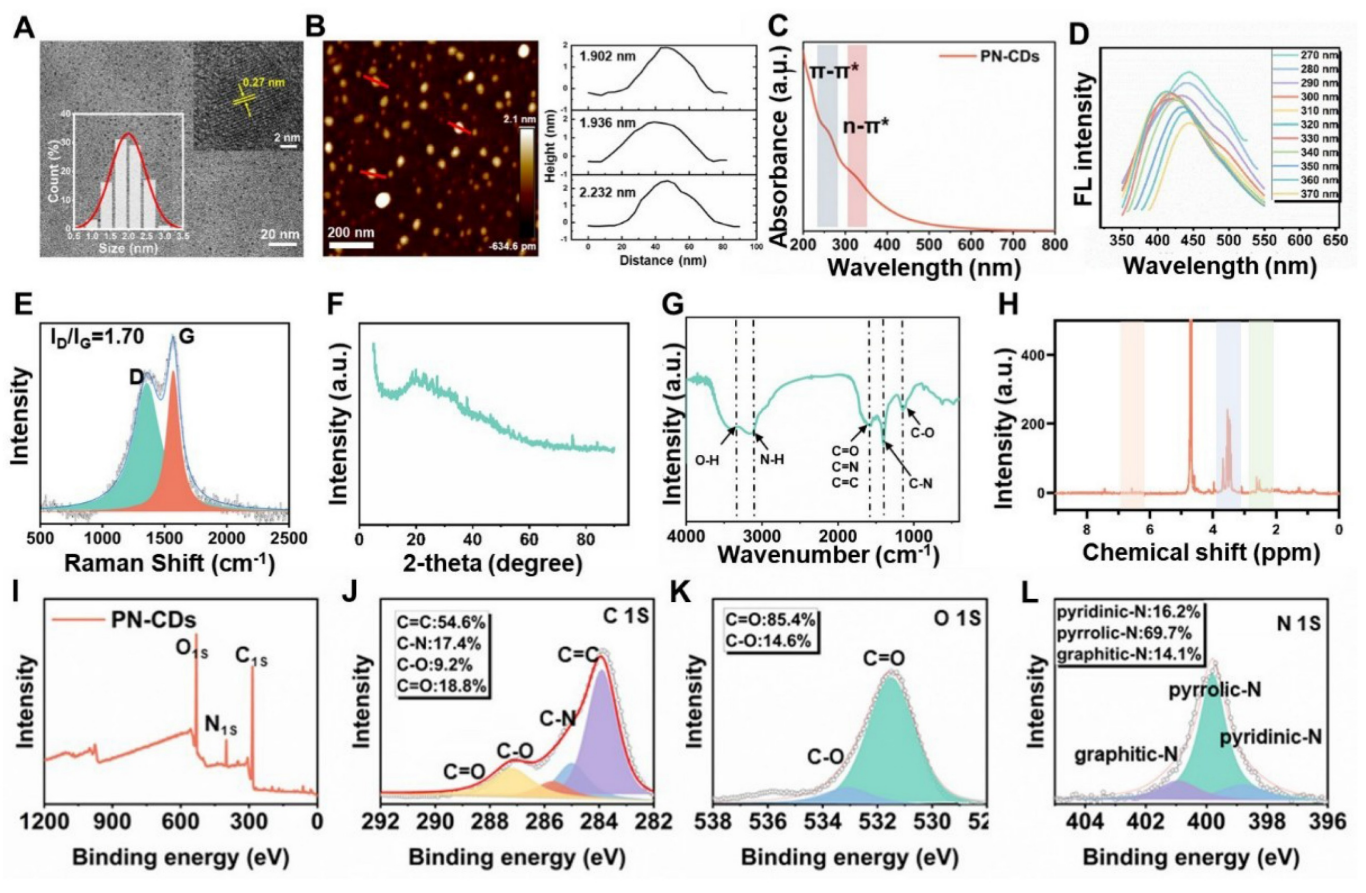
** Characterization of PN-CDs.** (A) Images of TEM and HRTEM, and size distribution histogram of PN-CDs. (B) AFM and distribution height of PN-CDs. (C) UV spectra of PN-CDs. (D) The fluorescence spectra of PN-CDs. (E) Raman spectrum of PN-CDs. (F) XRD pattern of PN-CDs. (G) FT-IR spectrum of PN -CDs. (H) ¹H-NMR spectrum of PN-CDs. (I) XPS survey spectrum of PN-CDs. High-resolution XPS spectra of C 1s (J), O 1s (K), and N 1s (L) for PN-CDs. TEM: Transmission electron microscopy; HRTEM: High-resolution transmission electron microscopy; AFM: Atomic force microscopy. XRD: X-ray diffraction. FT-IR: Fourier-transform infrared spectroscopy. XPS: X-ray photoelectron spectroscopy.

Using a hydrothermal technique, *Panax notoginseng* was utilized to produce PN-CDs, and transmission electron microscopy (TEM) analysis indicated they were spherical. According to the statistical evaluation, the average particle size was determined to be 2.0 nm, with a variation of ± 0.3 nm. High-resolution transmission electron microscopy (HRTEM) revealed that PN-CDs exhibit a crystalline structure featuring a lattice spacing of 0.27 nm, which matches the (100) facets of graphite **(Figure 2A)**. From the above result, it can be observed that the PN CDs have a crystalline graphitic structure. From AFM measurements, we find that the PN-CDs are 2.02±0.18nm high, which is in nice agreement with the average size, so they are roughly spherical as shown in **Figure 2B**. CDs are essentially characterized by photoluminescence. The UV-visible absorption spectrum of PN-CDs displayed two peak absorptions at 270 nm and 330 nm, corresponding to π-π* and n-π* transitions, respectively, as illustrated in **Figure 2C**. The presence of sp^2^ π-conjugated carbon structures is responsible for the π -π* transition^28^. When these regions absorb ultraviolet light, the π electrons in the π bond orbitals undergo transition to the π* orbitals. The n-π*transition of PN-CDs originates from oxygen-containing functional groups such as hydroxyl (-OH) and carbonyl^29^, ^30^. Photon absorption is required to excite n-electrons to the π*orbitals. PN-CDs fluorescence spectrum shows an excitation wavelength from 270-370nm, with a narrow emission peak between 412-450nm, and it has excitation-dependent fluorescence emission. The excitation wavelength deemed optimal was 270 nm, and the emission wavelength deemed optimal was 450 nm** (Figure 2D)**. These results confirm the successful synthesis of PN-CDs.

To study the surface structural features of PN-CDs, Raman spectroscopy, XRD, FT-IR and XPS. were carried out. The Raman spectra of PN-CDs showed carbonaceous material characteristics with the presence of D-band and G-band peaks at 1361.97cm⁻¹ and 1562.52cm⁻¹, respectively. The G-band at 1562. 52 cm⁻¹ denotes the in-plane vibrational modes of sp²-hybridized carbon atoms and offers knowledge regarding graphite-like structural attributes. On the other hand, D band at 1361.97 cm^-1^ is related to the structural disorder and defects. The peak-fitting analysis revealed a high ratio of I(D)/I(G) intensity of 1.7, which indicates that there are many structural defects on the surface of the CDs as seen in **Figure 2E.** These defects probably result from the incomplete graphitization when *Panax notoginseng* biomass carbonizes, as well as the hybrid structures brought in by surface functional groups. XRD analysis shows a relatively wide peak at 22.7 degrees, with a d-spacing of 0.393 nm, as shown in **Figure 2F** (larger than the standard 0.335nm d-spacing of graphite's (002) plane. This finding suggests that PN-CDs possess an amorphous structure similar to graphite, characterized by low crystallinity and a high concentration of surface functional groups. These groups can insert themselves between the graphite layers, expanding the layer spacing.

Structural analysis revealed that PN-CDs possess a hybrid structure comprised of a defective graphite-like framework and abundant surface functional groups. FT-IR spectroscopy identified surface moieties through characteristic peaks: a broad band at 3500-3200 cm⁻¹ for O-H/N-H stretching vibrations (hydroxyl/amine groups), peaks between 1700-1600 cm⁻¹ for C = O, (C = N), and C = C modes, and vibrations near 1300-1000 cm⁻¹ for C-O and C-N (oxygen- and nitrogen-containing functional groups) **(Figure 2G)**. ¹HNMR spectroscopy further elucidated the hybrid nature: aromatic/olefinic hydrogen signals (δ≈6-7 ppm) indicated sp²-conjugated domains; signals from hydrogens alpha to heteroatoms (δ≈3-6 ppm) confirmed surface polar groups (hydroxyl, carboxyl, amino) imparting hydrophilicity and reactivity; and aliphatic hydrogen signals (δ≈1.5-2.5 ppm) suggested alkyl chains/fragments **(Figure 2H)**. XPS analysis determined the elemental composition as C (66.25%), O (25.6%), and N (8.15%) **(Figure 2I)**. High-resolution spectra identified the chemical states with C1s displayed components for C = C (284.6 eV), C-N (285.7 eV), C-O (286.5 eV), and C = O (287.0 eV). **(Figure 2J)**; O1s elements for C-O (533.0 eV) and C = O (531.5 eV) **(Figure 2K)**; N1s contributions from graphitic N (400.9 eV), pyrrolic N (399.7 eV) and pyridinic N contributions (398.6 eV) **(Figure 2L)**. Collectively, this defective graphite framework, rich in surface amino, hydroxyl, carboxyl, carbonyl, and other functional groups derived from the *Panax notoginseng* precursor during carbonization, underpins the PN-CDs' hydrophilicity, reactivity, and potential for pharmacological and catalytic applications.

### 2.2 Examination of PN-CDs' antioxidant enzyme activity *in vitro*

**Figure 3:**
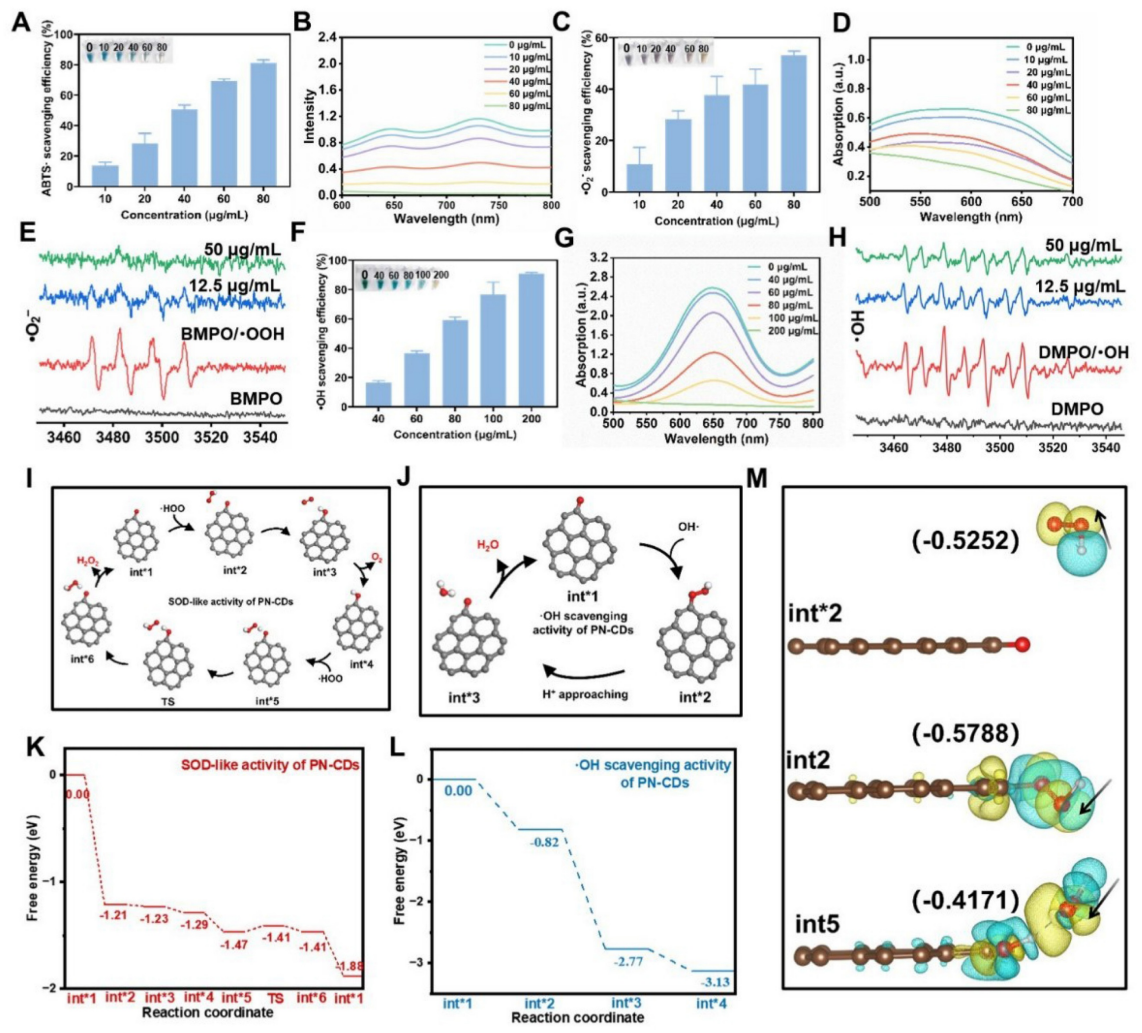
** Determination of the *in vitro* antioxidant capacity and free radical scavenging activity of PN-CDs** (A) 10-min elimination efficiency, (B) UV-Vis absorption spectra of •ABTS⁺ radicals under varying PN-CDs concentrations. (C) 10-min elimination efficiency (D) UV-vis absorption spectra, and (E) ESR signals for •O₂⁻ radical scavenging. (F) 10-min elimination efficiency (G) UV-vis absorption spectra, and (H) ESR signals for •OH radical scavenging. (I) Proposed reaction pathway and (K) Free energy diagram for the SOD-like catalytic cycle of PN-CDs. (J) Proposed reaction pathway and (L) Free energy diagram for the •OH scavenging catalytic cycle of PN-CDs. (M) The charge density of PN-CDs' three states with absorption-free energies was observed during the two catalytic cycles, with yellow and cyan indicating charge accumulation and depletion, respectively.

CDs have gained significant interest for their antioxidant properties and have been used in treating various diseases. The antioxidant enzyme activity of PN-CDs was examined through ABTS, NBT, and TMB assays to evaluate their ability to scavenge free radicals. The ABTS assay was initially used to assess the total antioxidant capacity of PN-CDs. ABTS was reacted with an oxidant overnight to produce the •ABTS⁺ radical. PN-CDs were mixed with •ABTS^+^ of different concentrations. Recorded absorbance of the solution at 734 nm every 1 min. The results indicated that an increment of PN-CDs' concentration gradually faded the mixture's blue color and decreased its absorbance, indicating the powerful antioxidant property of PN-CDs **(Figure 3A-B)**. The capacity of PN-CDs for scavenging •O₂⁻ was evaluated using NBT colorimetric tests, WST-1 commercial SOD assay kit, and ESR spectroscopy. In the NBT-based detection system, higher doses of PN-CDs cause a dose-dependent reduction in the absorbance at the characteristic wavelength of the •O₂⁻. NBT adduct **(Figure 3C-D)**. Furthermore, PN-CDs also showed a SOD-like activity of 598.3 U/mg** (Figure S1)**, thus suggesting the efficient scavenging ability of PN-CDs towards •O₂⁻. This scavenging activity was further verified by ESR with BMPO as the trap, and the BMPO-•OOH adducts diagnostic for •O₂⁻ trapping was significantly diminished by PN-CDs treatment, especially at high concentration** (Figure 3E)**. For•OH scavenging evaluation, TMB colorimetric assay was used TMB oxidation, which is very sensitive to •OH, showed a concentration-dependent inhibition in the presence of PN-CDs, implying their •OH scavenging activity **(Figure 3F-G)**. ESR spectroscopy directly confirmed this by employing DMPO as a spintrapping agent and the signal intensity of DMPO-OH adducts was obviously reduced after introducing PN-CDs, indicating that PN-CDs can scavenge •OH** (Figure 3H).**

Density functional theory (DFT) calculations have revealed the anti-oxidative mechanism of PN-CDs at the atomic level. Conjugated carbonyl groups in the π-system are very active functional groups in CDs nanozymes, which can serve as good active centers for SOD-like activity^31^. A model that carries carbonyls conjugated to the π system was synthesized to investigate the catalytic mechanisms underlying the SOD-like activity and •OH scavenging activity of PN-CDs. As a Brønsted base, •O_2_⁻ readily abstracts protons from the water to form •HOO. Then, the free •HOO will be adsorbed and initially binds to the carbonyl group to form an H-O bond. The carbonyl group subsequently oxidizes the •HOO radical, which leads to the creation of hydroxyl groups conjugated to the π-system (Int*2→ Int*4), and O₂ molecules are also formed. Additionally, the hydroxyl group within the molecule is able to absorb another second •HOO, which would cause the hydrogen abstraction and convert back to the original carbonyl structure with π-system conjugation. It results in H_2_O_2_molecules (Int* 5 → Int* 1), finishing the whole catalytic cycle **(Figure 3I)**. The reaction pathway has 7 states, and TS is a complex structure modification that allows PN-CDs do SOD-like cyclic catalysis. From the energy profile, it can be seen that the adsorption of •HOO between Int*1 and Int*2 has a great energy reduction (ΔE: -1.21eV), which indicates that the adsorption process of •HOO by PN-CDs is easy to happen and can proceed spontaneously **(Figure 3K)**.

Reaction mechanism of •OH scavenging by PN-CDs is given in **Figure 3J**. Firstly, the free •OH radical attaches to and chemically binds with the carbonyl group, producing the intermediate Int* 2. Then, the terminal oxygen atom of the •OH reacts with free protons (H⁺) in the surroundings to form H_2_O molecules. Lastly, the bond between the carbonyl oxygen and the hydrogen atom of the H_2_O molecule is cleaved and the H_2_O molecule is released from the active site, completing the whole process **(Figure 3J)**. By energy spectrum, we can see that the adsorption of •OH between Int*1 and Int*2 is spontaneous and exothermic; the energy change is -0.82 eV **(Figure 3L).** Additionally, the whole reaction is a decrease in energy, which shows that the presence of PN-CDs in the solution allows the spontaneous scavenging of •OH without external energy being added.

Under the condition of two catalytic cycles, the charge difference density of three states with adsorption free energy is shown **(Figure 3M)**. In Int*2, the oxygen atom of the absorbed hydroxyl is charged up, and therefore it can bind to H^+^ ions in the surroundings. Int*2 and Int*5 both show significant charge transfer around the carbonyls conjugated to the pi system, which is an important part of the catalytic process. This mechanistic study indicates that the carbonyl-containing functional groups in PN-CDs serve as active sites to facilitate enzymatic catalytic reactions.

### 2.3 PN-CDs cellular uptake and mitochondrial colocalization of PN-CDs

**Figure 4:**
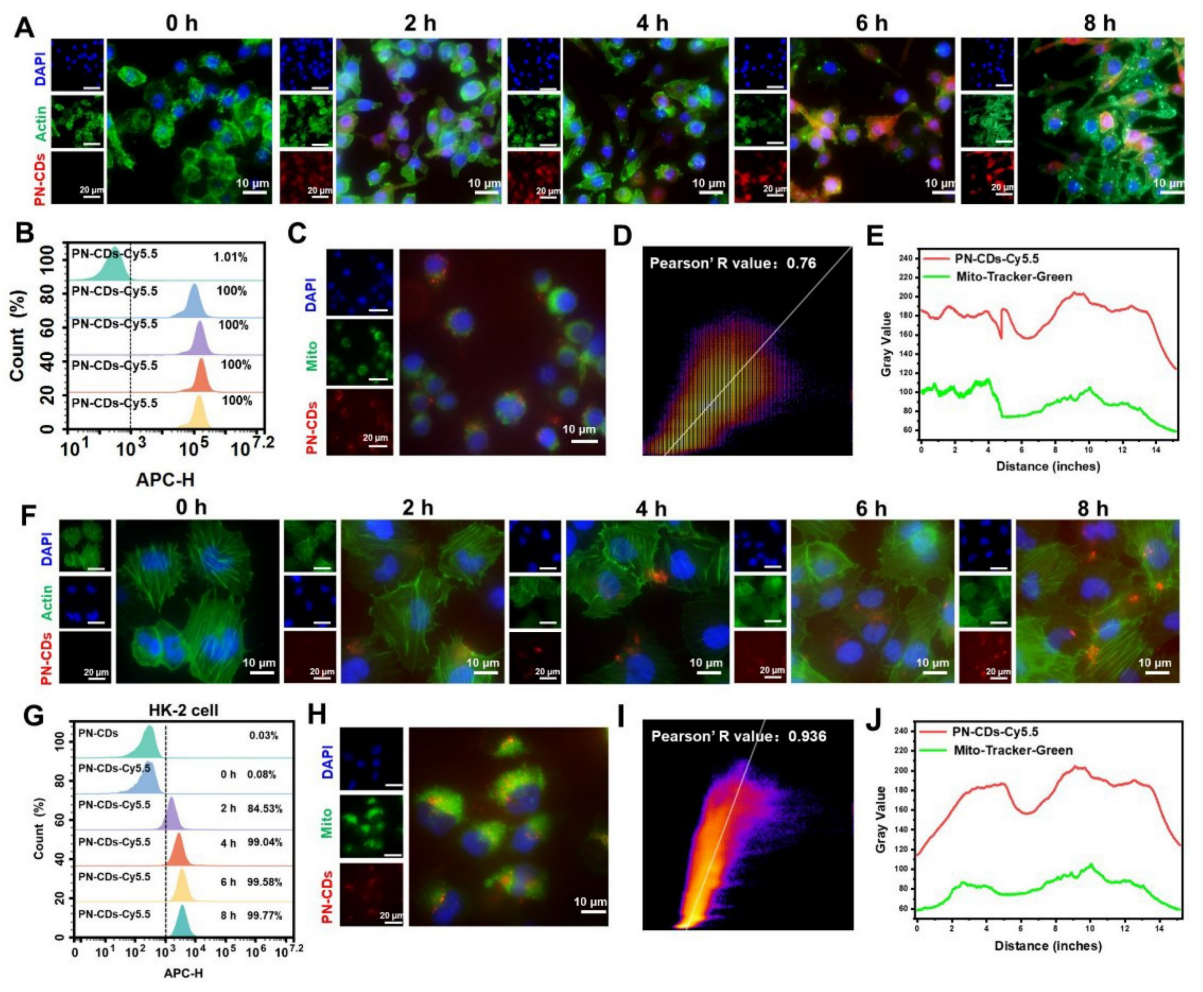
** Cellular uptake and mitochondrial colocalization of PN-CDs.** (A) Fluorescence images showing the time-dependent cellular uptake of PN-CDs in LPS-induced RAW 264.7 cells. Cells were stained with DAPI (blue, nuclei) and Actin (green, cytoplasm); PN-CDs were labeled with Cy5.5 (red). (B) Flow cytometry quantification of PN-CDs-Cy5.5 uptake efficiency in LPS-induced RAW 264.7 cells at different time points (0, 2, 4, 6, 8 h). (C) Fluorescence colocalization of PN-CDs (red) with mitochondria (green, labeled by Mito-Tracker Green) in RAW264.7 cells. Nuclei were stained with DAPI (blue). (D) Pearson's correlation coefficient analysis of PN-CDs and mitochondrial colocalization using Image J software. (E) Plot profile analysis of PN-CDs-Cy5.5 (red) and Mito-Tracker Green (green) fluorescence. (F) Fluorescence images showing the time-dependent cellular uptake of PN-CDs in HK-2 cells. (G) Flow cytometry quantification of PN-CDs-Cy5.5 uptake efficiency in HK-2 cells at different time points, with percentages of positive cells indicated. (H) Fluorescence colocalization of PN-CDs (red) with mitochondria (green) in HK-2 cells. (I) Pearson's correlation coefficient analysis of PN-CDs and mitochondrial colocalization in HK-2 cells. (J) Plot profile analysis of PN-CDs-Cy5.5 and Mito-Tracker Green fluorescence.

Efficient cellular uptake of PN-CDs is the prerequisite for exerting their anti-inflammatory and antioxidant effects. To track their intracellular distribution, PN-CDs were labeled with the red fluorescent probe Cy5.5. Fluorescence colocalization and flow cytometry results demonstrated that both normal RAW 264.7 cells and RAW 264.7 cells stimulated with LPS were capable of effectively internalizing PN-CDs. Specifically, in normal RAW 264.7 cells, the intracellular fluorescent signal intensity gradually augmented over time (**Figure S2**), and approximately 99.52% of PN-CDs were internalized after 8 hours of co-incubation (**Figure S3**). Nearly 100% of PN-CDs-Cy5.5 were internalized by LPS-induced RAW 264.7 cells within 2 hours of co-incubation **(Figure 4A-B)**. This phenomenon might be attributed to the fact that after macrophages are activated by LPS, they express more pattern recognition receptors on their surfaces, which promotes the phagocytosis of macrophages. Mitochondria are the primary source of ROS within cells, suggesting that the antioxidant effects of PN-CDs are mainly concentrated in these organelles. To investigate whether PN-CDs can enter mitochondria, mitochondria were labeled with the MitoTracker fluorescent probe. Fluorescence colocalization analysis indicated that PN-CDs were colocalized with green-labeled mitochondria, as shown in **Figure 4C**, with a Pearson correlation coefficient of 0.76 **(Figure 4D)**. The Pearson correlation coefficient is employed to quantify co-localisation phenomena, with values ranging from -1 to 1. A coefficient of 1 indicates a perfect positive correlation between the fluorescence intensities of two images, whereas a value of -1 signifies a perfect negative correlation^32^. Plot profile analysis further showed that the fluorescence signal peaks of PN-CDs-Cy5.5 and mitochondria were largely consistent **(Figure 4E).**

To continue the investigation on the uptake and mitochondrial colocalization of PN-CDs in renal tubular epithelial cells, similar experiments were carried out in HK-2 cells. Fluorescence imaging and flow cytometry results indicate that HK-2 cells exhibit increased uptake of PN-CDs over time, as shown in **Figure 4F**, and 99.77% of PN-CDs were internalized after 8h, as shown in **Figure 4G**. In the mitochondrial colocalization assay in HK-2 cells, it was observed that PN-CDs targeted to the mitochondria of HK-2 cells, and the Pearson correlation coefficient was 0.936 **(Figure 4H-I)**. As expected, the plot profile showed overlapping fluorescence signal peaks of PN-CDs-Cy5.5 and mitochondria **(Figure 4J)**. Collectively, these results show that different cell types are able to internalize PN-CDs and specifically target intracellular mitochondria. Such properties enable PN-CDs to efficiently scavenge ROS at the primary site of their production in cells.

### 2.4 Anti-inflammatory and antioxidative stress activity of PN-CDs at the cellular level

**Figure 5:**
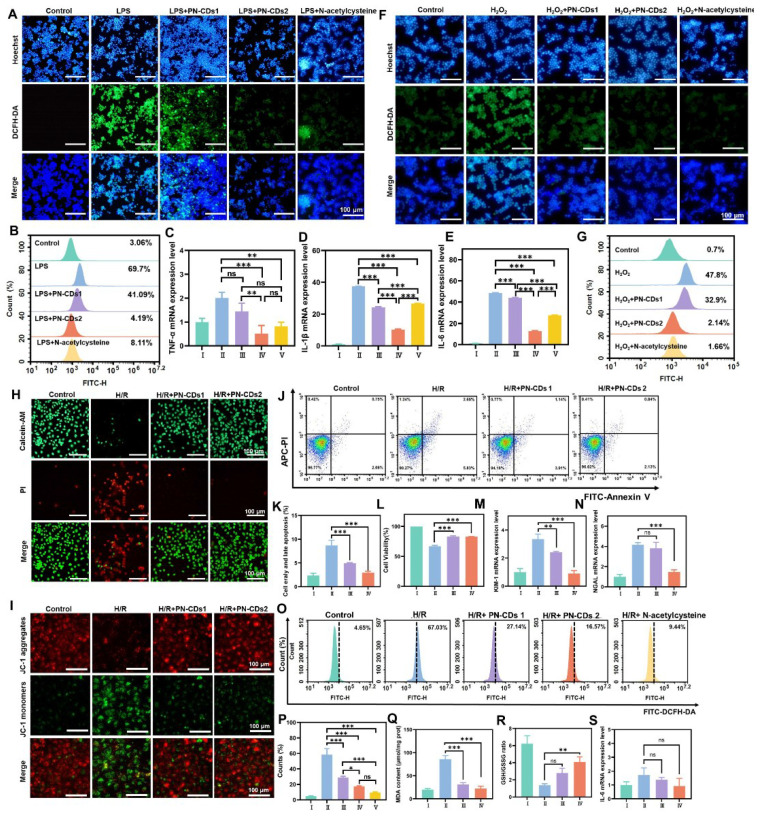
** Anti-inflammatory and antioxidative stress activity of PN-CDs at the cellular level.** (A) Fluorescence images showing intracellular ROS levels (green, DCFH-DA) and nuclear staining (blue, Hoechst) in RAW 264.7 cells treated with control, LPS, LPS+ PN-CDs1 (5 μg/mL), LPS + PN-CDs2 (10 μg/mL), or LPS+ N-acetylcysteine. (B) Flow cytometry analysis of intracellular ROS levels in RAW 264.7 cells under the above treatments. qPCR analysis of pro-inflammatory cytokine mRNA expression in LPS-induced RAW 264.7 cells. (C) TNF-α, (D) IL-1β, (E) IL-6. I: Control; II: LPS; III: LPS + PN-CDs1; IV: LPS + PN-CDs2, Ⅴ: LPS+ N-acetylcysteine. Data are presented as mean ± SD. Statistical significance: ***p < 0.001 vs. LPS group; ns: not significant. (F) Confocal fluorescence images showing intracellular ROS levels (green, DCFH-DA) and nuclear staining (blue, Hoechst) in RAW 264.7 cells treated with control, H₂O₂, H₂O₂ + PN-CDs1, H₂O₂ + PN-CDs2, or H_2_O_2_+ N-acetylcysteine. (G) Flow cytometry analysis of intracellular ROS levels (DCFH-DA fluorescence, FITC-H channel) in H₂O₂-induced RAW 264.7 cells. (H) Calcein/PI fluorescence staining showing live cells (green, calcein) and dead/apoptotic cells (red, PI) in Control, H/R, H/R + low-concentration PN-CDs (PN-CDs1), and H/R + high-concentration PN-CDs (PN-CDs2) groups. (I) JC-1 mitochondrial membrane potential (ΔΨm) assay: Red fluorescence indicates J-aggregates (high ΔΨm), green fluorescence indicates monomers (low ΔΨm), and Merge images overlay red/green signals. Annexin V-FITC/PI flow cytometry analysis of apoptosis: (J) Representative scatter plots showing viable (Q4), early apoptotic (Q3), late apoptotic (Q2), and necrotic (Q1) cells; (K) Quantitative analysis of total apoptotic cells (Q2 + Q3). (L) CCK-8 assay showing cell viability in each group. qPCR analysis of renal tubular injury markers: (M) Kidney injury molecule-1 (KIM-1) and (N) neutrophil gelatinase-associated lipocalin (NGAL) mRNA expression. Flow cytometry analysis of intracellular ROS levels (DCFH-DA fluorescence, FITC channel): (O) Representative histograms; (P) Quantitative analysis of ROS-positive cells. Biochemical analysis of oxidative stress markers: (Q) Malondialdehyde (MDA) levels and (R) The ratio of glutathione (GSH)/glutathione (GSSG). (S) qPCR analysis of IL-6 mRNA expression in H/R model. Data are presented as mean ± SD. Statistical significance: **p < 0.01, ***p < 0.001 vs. H/R group; ns, not significant. I: Control; II: H/R; III: H/R + PN-CDs1; IV: H/R + PN-CDs2, Ⅴ: H/R+ N-acetylcysteine.

Elevated inflammation and oxidative stress are key pathological features of RIRI. Based on the *in vitro* antioxidant activity of PN-CDs, we further evaluated their anti-inflammatory and antioxidant effects at the cellular level. Specifically, we used LPS and H₂O₂ to induce inflammation and oxidative stress in RAW 264.7 cells, respectively, and then assessed the impact of PN-CDs on these processes. 2',7'-Dichlorodihydrofluorescein diacetate (DCFH-DA) serves as a probe for detecting ROS within cells. Upon oxidation, this compound generates the green fluorescent compound 2',7'-dichlorofluorescein (DCF). The fluorescence imaging revealed a significant increase in green fluorescence intensity within cells in the LPS-induced group, indicating elevated levels of ROS. In contrast, both low-concentration (5 μg/mL, PN-CDs1) and high-concentration (10 μg/mL, PN-CDs2) PN-CDs markedly reduced this fluorescence intensity **(Figure 5A).** Flow cytometry analysis showed that LPS stimulation greatly increased the intracellular ROS level, while PN-CDs1 and PN-CDs2 treatment considerably decreased ROS level and showed effects comparable to the positive control, N-acetylcysteine **(Figure 5B)**. To evaluate PN-CDs' anti-inflammatory activity, we determined pro-inflammatory cytokine expression by quantitative real-time polymerase chain reaction (qPCR). Anti-inflammatory effects of PN-CDs were assessed by quantification of pro-inflammatory cytokines expression via quantitative real-time qPCR. The study demonstrated that the PN-CDs1 and PN-CDs2 significantly decreased LPS-induced TNF-α, IL-1β, and IL-6 levels expressed in LPS-stimulated RAW 264.7 cells, as shown in **Figures 5C, 5D, and 5E**. All data collectively indicate that PN-CD reduces LPS-induced inflammatory responses by inhibiting the production of pro-inflammatory cytokines.

Next, we employed a hydrogen peroxide-induced oxidative stress model utilising RAW 264.7 cells to validate the antioxidant activity of PN-CDs. DCFH-DA and dihydroethidium (DHE) were employed to measure total oxidant activity (ROS) and •O₂⁻ within cells, respectively. In confocal fluorescence imaging, H₂O₂ treatment significantly increased the green fluorescence intensity of ROS** (Figure 5F)** and •O₂⁻ **(Figure S4)** compared with the control group. Flow cytometry analysis also supported these results, revealing that the H₂O₂-induced cells had an increased level of total ROS and this was reduced by PN-CDs **(Figure 5G)**. Together, these results show that both low and high concentrations of PN-CDs have attenuated LPS-induced inflammation and reduced the ROS level in RAW 264.7 cells. This data support the ability of PN-CDs to attenuate cellular oxidation and inflammation, necessary for PN-CDs' potential use in RIRI.

To investigate the effect of PN-CDs on RIRI, a standard HK-2 cell H/R model was established to mimic ischemia/reperfusion injury. Effects of PN-CDs on cell apoptosis and proliferation were detected by Calcein/PI staining, JC-1 mitochondrial membrane potential detection, Annexin V apoptosis detection kit, and CCK-8 detection. Calcein/PI fluorescence staining showed that the H/R group greatly promoted cell apoptosis, but both low and high concentrations of PN-CDs greatly reduced the number of apoptotic cells** (Figure 5H).** Depolarization of the mitochondrial membrane potential (ΔΨm) indicates the occurrence of early apoptosis and mitochondrial damage. JC-1, a cationic fluorescent probe, forms J-aggregates with red fluorescence in the mitochondrial matrix at high ΔΨm, but dissociates to green fluorescent monomers at low ΔΨm. JC-1 staining showed that the H/R group had an obvious increase in green fluorescence intensity (representing ΔΨm depolarization), and PN-CDs inhibited the loss of H/R ΔΨm, which was reflected in the increased red fluorescence intensity of J-aggregates **(Figure 5I)**. Annexin V flow cytometry revealed that H/R significantly elevated early and late apoptosis cells, which PN-CDs reversed **(Figure 5J-K)**. The CCK-8 assay further confirmed that H/R reduced cell viability, while PN-CDs significantly improved viability **(Figure 5L).** Neutrophil gelatinase-associated lipocalin (NGAL) and kidney injury molecule-1 (KIM-1) transcript levels, indicators of renal tubular cell injury, exhibited significant elevation in the H/R group but decreased following PN-CDs treatment **(Figure 5M-N)**.

Since H/R models typically increase intracellular ROS, inflammation, and lipid peroxidation, we measured intracellular ROS levels via flow cytometry, which showed that H/R-induced ROS elevation was significantly suppressed by PN-CDs, exhibiting a comparable effect to the positive control N-acetylcysteine **(Figure 5O-P)**. Additionally, H/R increased malondialdehyde (MDA) levels and decreased the ratio of glutathione (GSH)/glutathione (GSSG) levels, whereas PN-CDs reversed these changes by reducing MDA and increasing GSH/GSSG **(Figure 5Q-R)**. qPCR analysis revealed no significant trends in pro-inflammatory cytokines, possibly due to minimal changes induced by H/R in the HK-2 cell model **(Figure 5S)**. Therefore, to further investigate the anti-inflammatory role of PN-CDs in kidney tubular cells, HK-2 cells induced by LPS were employed. qPCR analysis revealed that PN-CDs down-regulated TNF-α and IL-1β mRNA expressions, while IL-6 mRNA expression remained unaffected **(Figure S5A-C)**. These findings suggest that PN-CDs exert certain anti-inflammatory and antioxidant effects in renal tubular epithelial cells.

### 2.5 *In vivo* distribution of PN-CDs

**Figure 6:**
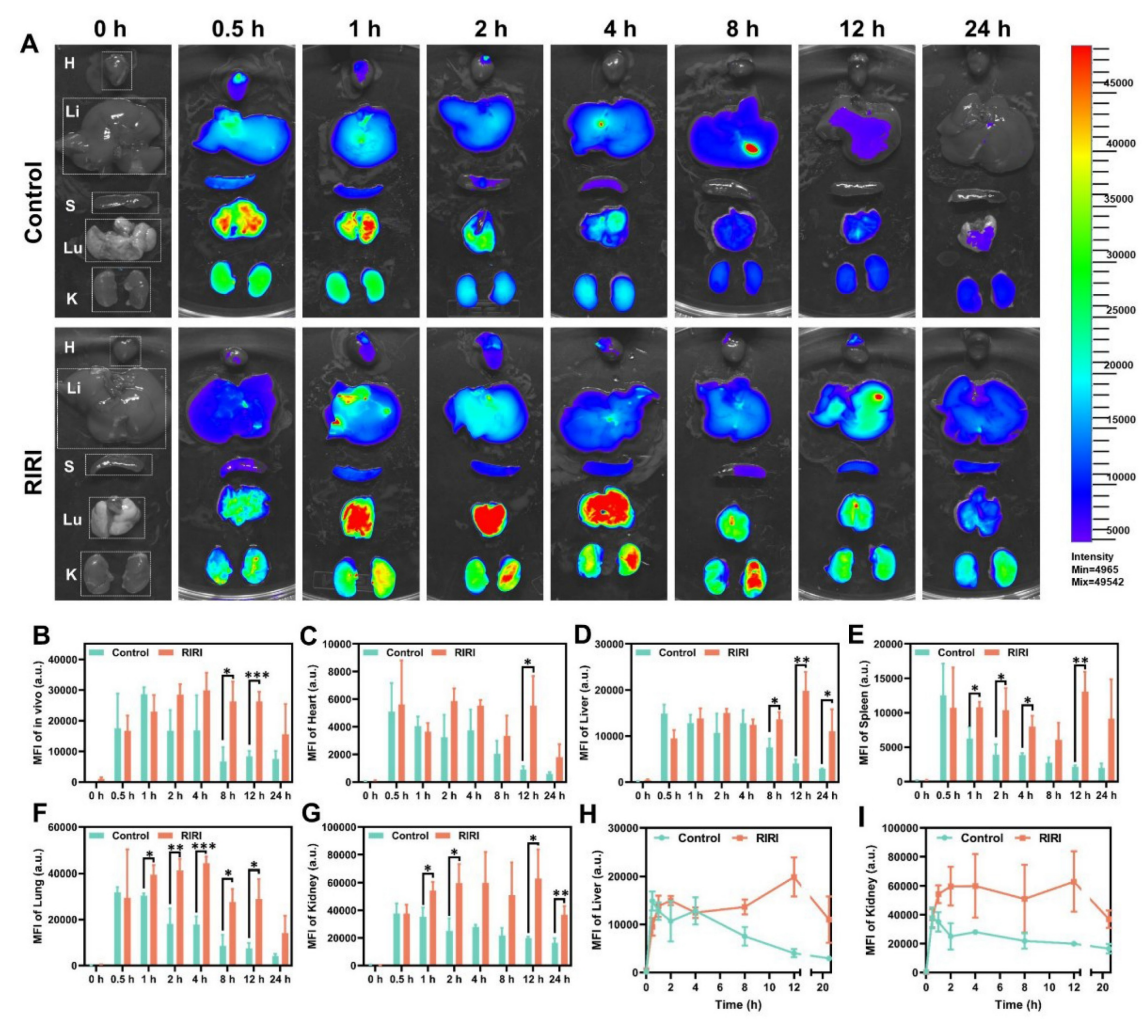
***In vivo* biodistribution and retention of PN-CDs-Cy5.5.** (A). *Ex vivo* organ fluorescence imaging of PN-CDs-Cy5.5 in control and RIRI model mice at 0 h, 0.5 h, 1 h, 2 h, 4 h, 8 h, 12 h, and 24 h post-intraperitoneal injection. Insets show magnified views of major organs: heart (H), liver (Li), spleen (S), lung (Lu), and kidney (K). (B-G) Quantitative analysis of mean fluorescence intensity (MFI) of PN-CDs-Cy5.5 in whole-body (B) and major organs, including heart (C), liver (D), spleen (E), lung (F), and kidney (G), at different time points. Data are presented as mean ± SD (n = 3 per group). *p < 0.05, **p < 0.01, ***p < 0.001 vs. Control group. (H-I) Time-course profiles of MFI for liver (H) and kidney (I) in Control and RIRI groups, highlighting the delayed clearance and prolonged retention of PN-CDs-Cy5.5 in RIRI model mice.

Before applying PN-CDs *in vivo*, we conducted a comprehensive evaluation of their biodistribution and metabolic kinetics using near-infrared fluorescence imaging. To facilitate *in vivo* visualization, PN-CDs were conjugated with Cy5.5, a near-infrared fluorophore. PN-CDs-Cy5.5 was intraperitoneally administered to both healthy and RIRI mice, and their dynamic biodistribution was evaluated at multiple time points (0, 0.5, 1, 2, 4, 8, 12, 24 hours) using whole-body and *ex vivo* organ imaging. Whole-body fluorescence imaging indicated that in healthy controls, the fluorescence of PN-CDs-Cy5.5 peaked at 1 hour post-injection. In contrast, RIRI models exhibited a delayed peak fluorescence at 4 hours post-injection, with a significantly slower signal decay rate compared to controls. Notable differences were observed at 8 and 12 hours **(Figure 6A-B, Figure S6)**. The quantitative analysis revealed that in the healthy controls, PN-CDs accumulated significantly in vital organs such as the heart, liver, spleen, lungs, and kidneys at 0.5 hours after injection and then began to decrease. In RIRI models, the peak accumulation was delayed to 12 hours post-injection for most organs, with the exception of lungs, which peaked at 4 hours **(Figure 6C-H)**. Renal enrichment of PN-CDs-Cy5.5 was most prominent in RIRI models **(Figure 6G)**. The kidney tissues kept considerably more fluorescence signals than controls throughout the time** (Figure 6I)**. These results show that the RIRI environment noticeably slows down how quickly PN-CDs are used up and removed from the body, not just all over but also inside different parts of the body. PN-CDs exhibit preferential passive accumulation in injured kidneys, a property that may facilitate targeted therapy for RIRI. The underlying mechanism may stem from the enhanced permeability and retention (EPR) effect observed in inflammatory tissues within the RIRI model. At the injury site, vascular hyperpermeability and endothelial barrier disruption occur^33^. Meanwhile, lymphatic function in the damaged renal area is impaired, leading to increased interstitial fluid pressure and PN-CDs retention^34^. Simultaneously, in the inflammatory microenvironment of the RIRI model, acute-phase proteins in the bloodstream bind to PN-CD surfaces, forming a protein corona that reduces macrophage recognition and phagocytic clearance^35^. Furthermore, in the RIRI model, the glomerular filtration rate is decreased, and tubular reabsorption and secretion functions are impaired, which prolongs the systemic circulation time of PN-CDs.

### 2.6 Renoprotective effect of PN-CDs in the RIRI model

**Figure 7:**
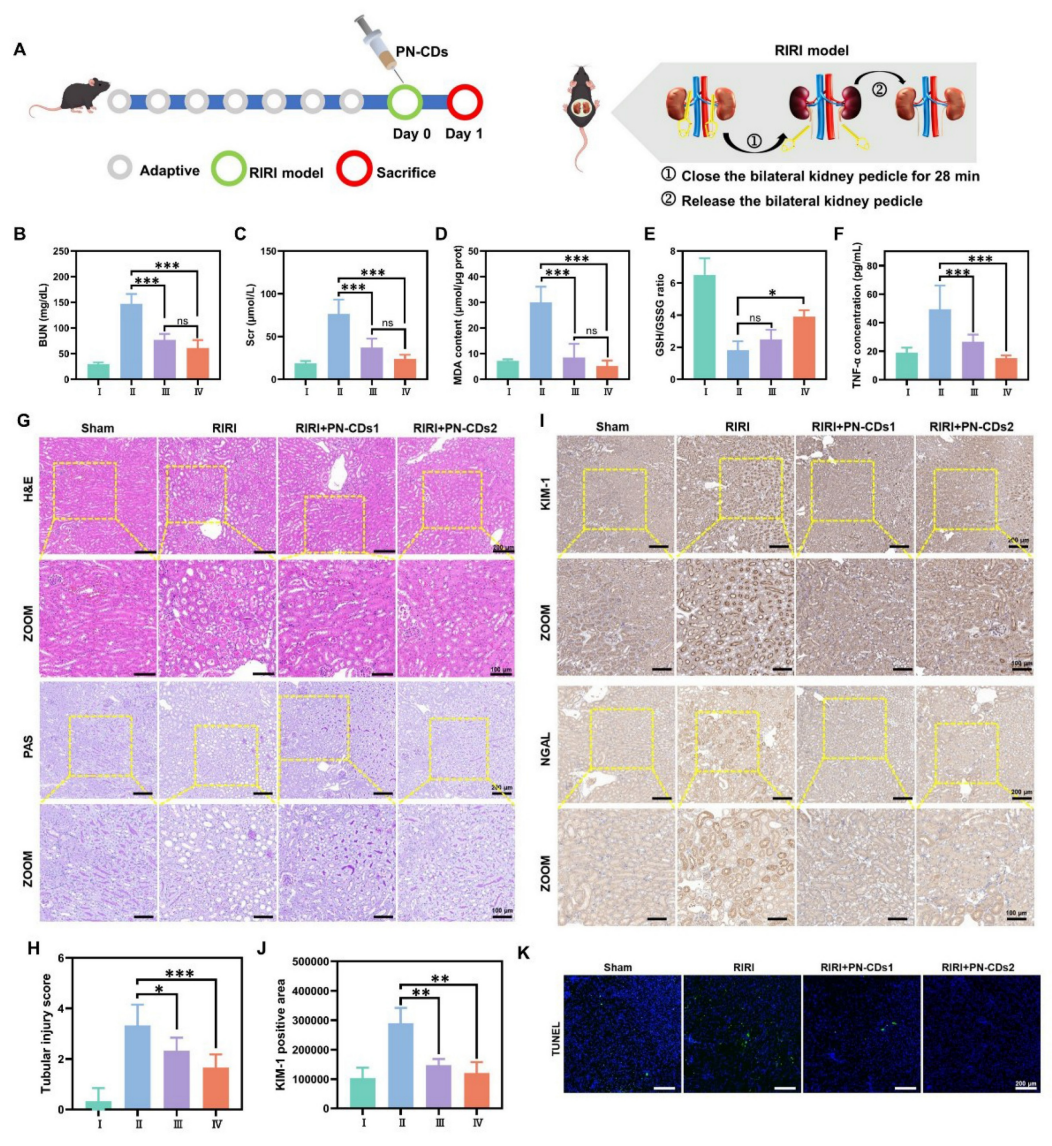
** PN-CDs mitigate RIRI in mice.** (A) Schematic of the experimental design. (B-F) Biochemical assessments of renal function and injury: (B) Blood urea nitrogen (BUN), (C) serum creatinine (Scr); (D) malondialdehyde (MDA), (E) GSH/GSSG ratio; (F) Tumor necrosis factor-α (TNF-α). (G) Histological analysis via H&E and PAS staining. (H) Tubular injury scores quantified from H&E-stained sections. (I) Immunohistochemical staining for kidney injury molecule-1 (KIM-1) and neutrophil gelatinase-associated lipocalin (NGAL). (J) Quantification of KIM-1-positive areas from immunohistochemistry. (K) TUNEL staining to detect apoptotic cells (green signal). Data are mean ± SEM; *p < 0.05, **p < 0.01, ***p < 0.001 vs. RIRI group. I: Sham; II: RIRI; III: RIRI + PN-CDs1; IV: RIRI + PN-CDs2.

We employed a 28-minute bilateral renal pedicle occlusion-induced RIRI mouse model to evaluate the renal protective effects of PN-CDs, which possess anti-inflammatory and antioxidant properties. Mice received a single intraperitoneal injection of either low (1 mg/kg, PN-CDs1) or high (5 mg/kg, PN-CDs2) doses of PN-CDs **(Figure 7A)**. After 24 hours of treatment, renal function, oxidative stress, inflammation, histopathology, and apoptosis were evaluated. The RIRI model group exhibited significant renal impairment, manifested by elevated blood urea nitrogen (BUN) and serum creatinine (Scr) levels compared to the sham-operated control group. Notably, both PN-CDs1 and PN-CDs2 significantly mitigated these elevations, suggesting improved renal function **(Figures 7B-C)**. The RIRI model had higher MDA, a marker of lipid peroxidation **(Figure 7D)**, and disrupted redox balance (lowered GSH/GSSG ratio; **Figure 7E)**. Administration of PN-CDs at both doses improved these oxidative stress parameters, indicating their antioxidant capability. Inflammation is a sign of RIRI. Among all tested cytokines, TNF-α expression was increased in the RIRI group compared to the sham, and both PN-CDs doses downregulated TNF-α levels, indicating an anti-inflammatory effect** (Figure 7F).** qPCR results showed that PN-CDs downregulated IL-6 mRNA levels and upregulated IL-10 mRNA levels, shifting pro-inflammatory to anti-inflammatory cytokine profiles **(Figure S7A-B)**.

Histopathological examination using haematoxylin and eosin (H&E) and periodic acid-Schiff (PAS) staining revealed severe tubular injury in the RIRI group, accompanied by tubular epithelial cell oedema, wrinkling of the basement membrane, disappearance of the brush border, cavitation of tubules and infiltration of inflammatory cells. On the contrary, PN-CDs treatment remarkably recovered renal tissue architecture **(Figures 7G-H and Figure S8A)**. KIM-1 and NGAL, tubular injury biomarkers, had high expression in the RIRI group that was notably decreased by both PN-CDs doses, indicating attenuated renal injury **(Figures 7I-J, Figure S8B)**. TUNEL staining showed a considerable rise in the number of apoptotic cells in the RIRI group, yet PN-CDs administration substantially decreased the amount of apoptosis, pointing to an inhibition of cell death **(Figure 7K, Figure S8C)**. These findings suggest that PN-CDs, at both low and high doses, enhance renal function and mitigate renal injury and apoptosis caused by RIRI, providing renoprotective effects against ischemia-reperfusion injury.

### 2.7 Modulation of gut microbiota by PN-CDs in RIRI

**Figure 8:**
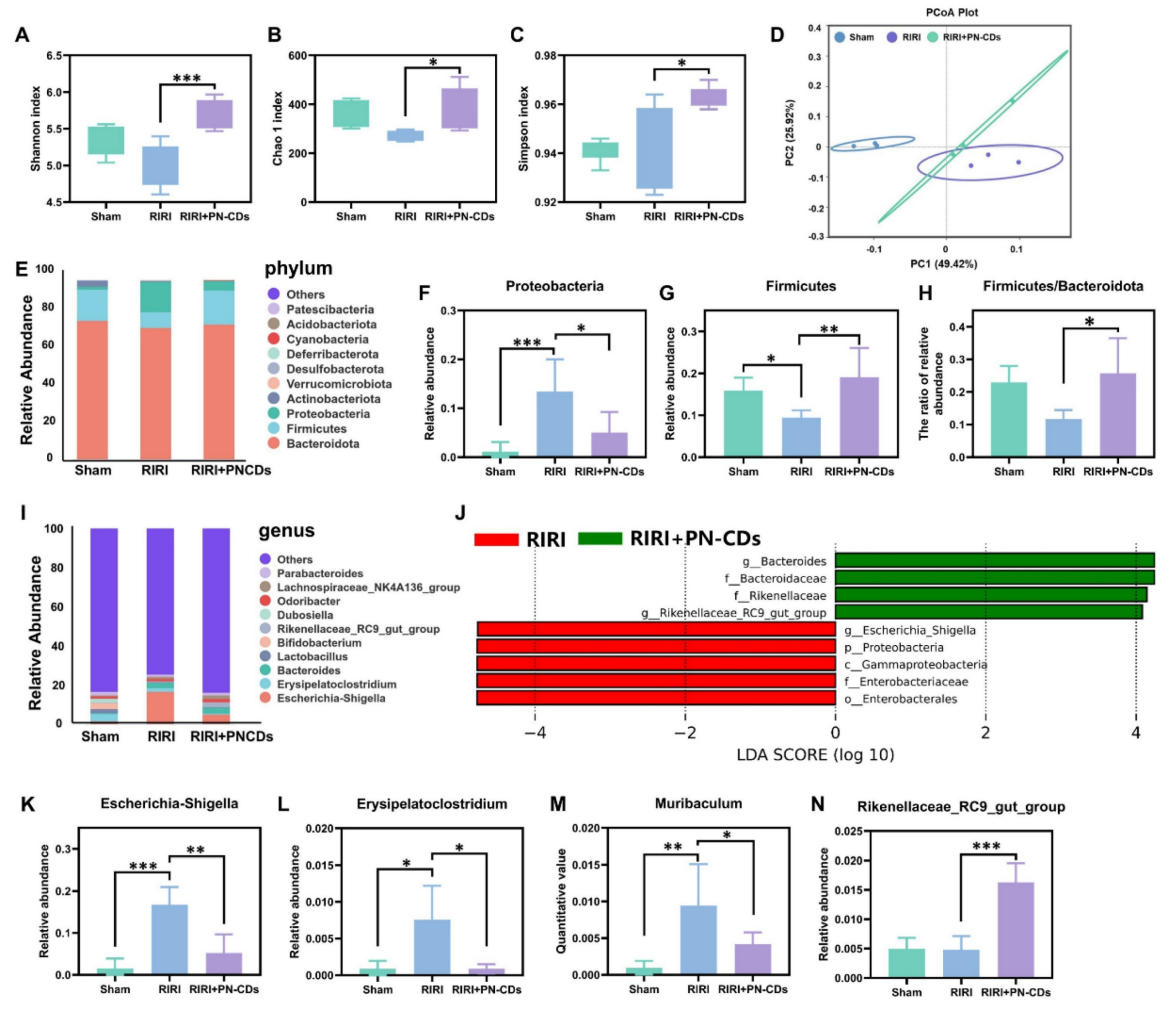
** Gut microbiota composition analysis in RIRI mice following PN-CDs treatment.** α-diversity characterization using the Shannon index (A), Chao1 index (B), and Simpson index (C) to compare gut microbial diversity/richness among Sham, RIRI, and RIRI+PN-CDs groups. (D) Principal component analysis (PCA) visualizing β-diversity differences in gut microbiota composition across experimental groups. (E) The relative abundance of dominant bacterial phylum in each group. (F) Relative abundance of the *Proteobacteria* phylum. (G) Relative abundance of the *Firmicutes* phylum. (H) The *Firmicutes/Bacteroidota* ratio. (I) The relative abundance of the top 10 dominant genera. (J) Linear discriminant analysis effect size (LEfSe) analysis identifying genera with differential abundance between RIRI and RIRI+PN-CDs groups (LDA score > 4). (K-N) Relative abundance of *Escherichia-Shigella* (K), *Erysipelatoclostridium* (L), *Muribaculum* (M), and *Rikenellaceae_RC9_gut_group* (N). Statistical significance was determined by one-way ANOVA with Tukey's post hoc test; *P < 0.05, **P < 0.01, ***P < 0.001.

Growing evidence has linked gut microbiota imbalance to the development of RIRI. Notably, PN-CDs have been suggested to modulate the abundance and structure of the gut microbiota. We analysed the gut microbiota in mouse feces samples under different treatment conditions using 16S rRNA gene sequencing technology, aiming to clarify the mechanistic role of PN-CDs in addressing RIRI. α diversity, which reflects the number of microbial species and their proportional distribution within a single sample, was assessed using three classic indices: the Shannon index (for species diversity), the Chao1 index (for species richness), the Simpson index (for the dominance of predominant species) and Observed Features (for species richness). **Figures 8A-C** demonstrate that the RIRI model group did not show a significant decrease in these indices, whereas the PN-CDs group significantly improved the Shannon, Chao1, and Simpson indices. However, no differences in Observed Features were observed among the three groups** (Figure S9A)**. It implied that PN-CDs raised the variety and quantity of gut microflora. Principal component analysis (PCA) was employed to assess β-diversity, a metric used to measure microbial community similarity across different samples. The PCA plot revealed distinct separation between the RIRI group and the sham surgery group, whilst the PN-CDs treatment group also exhibited significant divergence from the RIRI group **(Figure 8D)**. This suggested there were distinct gut microbiota of the three groups.

In order to get an idea of what the particular taxonomic breakdown was of RIRI, we looked at both the gut microbiota of RIRI by phyla and genus. The phylum-level composition of the gut microbiota is primarily comprised of the phyla *Firmicutes*, *Bacteroidetes*, and *Proteobacteria* **(Figure 8E)**. The RIRI group increased Proteobacteria significantly (Figure 8F) and reduced Firmicutes relative to the sham group** (Figure 8G)**. The PN-CDs group reduced both phyla significantly **(Figure 8H)**. No significant differences in Bacteroidetes abundance were observed across the three groups **(Figure S9B)**. Further analysis of the well-known Firmicutes/Bacteroidota ratio (the lower, the more dysbiosis) showed that it was lower in the RIRI group, but returned to a normal state by PN-CDs treatment **(Figure 8H)**. This means that PN-CDs could change the gut microbiota by making more of a group called Firmicutes and less of a group called Proteobacteria.

At the genus level, we considered the top 10 most abundant ones. The Sham group was enriched with *Erysipelatoclostridium*,* Lactobacillus,* and *Bifidobacterium*, RIRI group was enriched with* Escherichia-Shigella*, *Erysipelatoclostridium,* and *Bacteroides* **(Figure 8I)**. The Linear discriminant analysis effect size (LEfSe) was used to identify genera that were significantly different between the RIRI and PN-CDs group at the OTU level **(Figure 8J)**. 4 more key genera are further analyzed here: *Escherichia-Shigella*,* Erysipelatoclostridium*,* Muribaculum*,* and Rikenellaceae_RC9_gut_group*. Statistical results showed that the RIRI group had significantly more *Escherichia-Shigella*, *Erysipelatoclostridium*, and *Muribaculum* than the sham group, which were obviously decreased after PN-CDs treatment **(Figure 8K-M)**. There was no significant difference in the abundance of *Rikenellaceae_RC9_gut_group* between the sham-surgery group and the RIRI group; however, PN-CDs treatment significantly increased the abundance of this community **(Figure 8N)**. *Escherichia-Shigella* abundance is strongly and positively correlated with serum indoxyl sulfate levels^36^, uremic toxin associated with the decline in renal function. Significantly, much accumulated evidence from many studies has found a strong connection between E. shigella overgrowth and the development of different kidney disease^37^, ^38^. This bacterial genus is a significant risk factor for vascular calcification, a prevalent and serious complication in chronic kidney disease patients^39^.

Our results combined with existing research show that RIRI greatly changed gut microbiota composition and quantity, but PN-CDs administration helped to return the microbial structure and composition back. More research is necessary to see whether PN-CDs improve kidney damage by changing gut bacteria-generated uremic poisons.

### 2.8 PN-CDs alleviate RIRI by regulating the tryptophan-indole-AHR/ERK pathway

**Figure 9:**
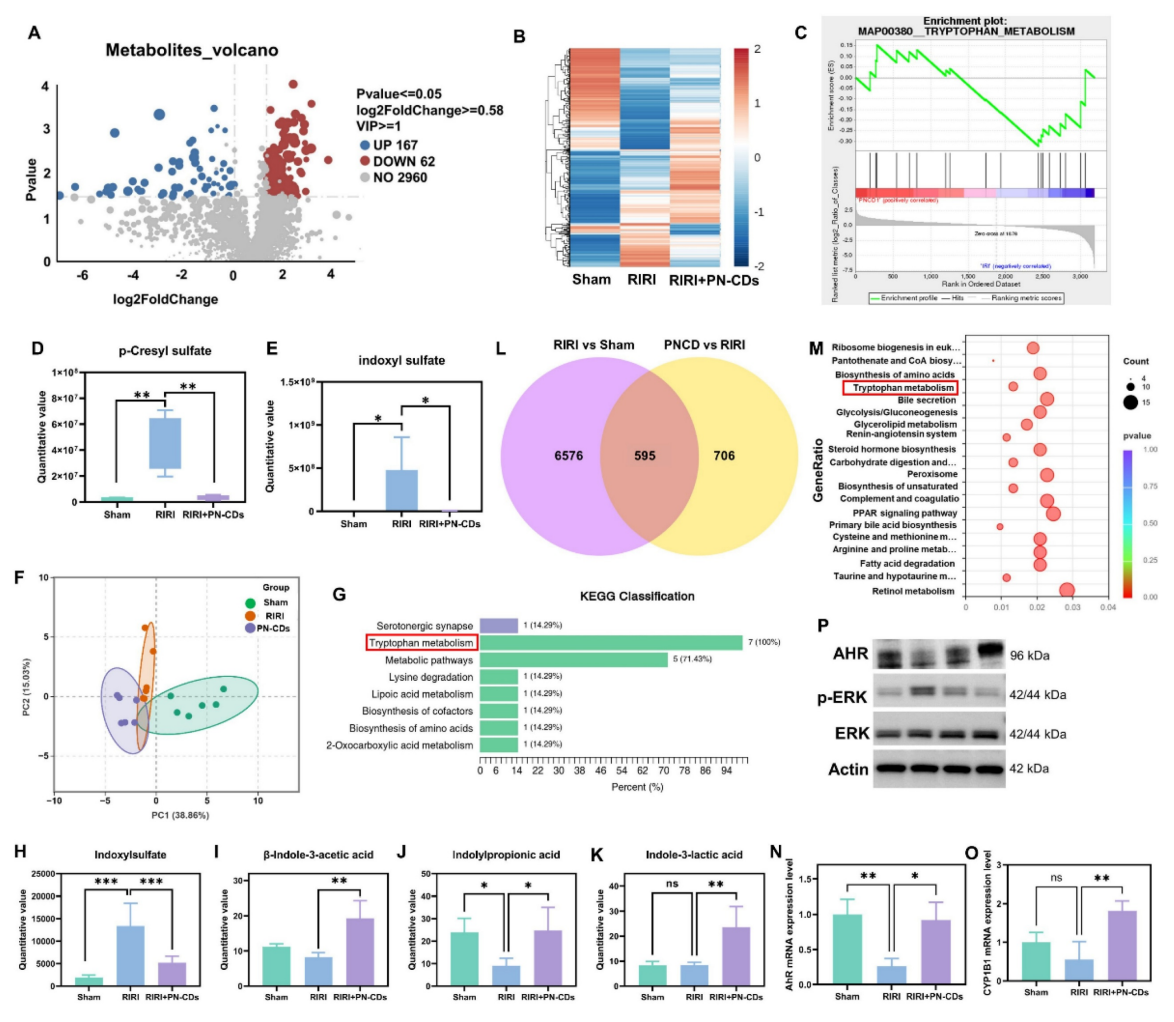
** Multi-omics investigation into the mechanism by which PN-CDs alleviate renal injury.** (A) Volcano plot of fecal metabolites comparing PN-CDs vs. RIRI groups; red/blue dots indicate significantly upregulated (167) or downregulated (62) metabolites (P < 0.05, VIP > 1, |log2FC| > 0.58). (B) Hierarchical clustering of differential metabolites, demonstrating distinct metabolic profiles across Sham, RIRI, and PN-CDs groups. (C) GSEA enrichment plot. (D-E) Quantification of uremic toxins p-cresyl sulfate (D) and indoxyl sulfate (E) in feces. (F) PCA of targeted tryptophan metabolites. (G) KEGG enrichment of targeted metabolites. (H-K) Quantification of key tryptophan-indole metabolites: indoxyl sulfate (H), β-indole-3-acetic acid (I), indolylpropionic acid (J), and indole-3-lactic acid (K). (L) Venn diagram of differential genes between RIRI vs. Sham and PN-CDs vs. RIRI. (M) Bubble plot of KEGG enrichment for overlapping genes, highlighting tryptophan metabolism and inflammatory pathways. (N-O) qPCR analysis of AHR (N) and CYP1A1 (O) mRNA levels in kidneys. (P) Western blot of AHR, p-ERK, ERK, and Actin in renal tissues. Data are mean ± SEM (ns, not significant, **P < 0.01, ***P < 0.001).

To elucidate whether PN-CDs confer renal protection by suppressing gut microbiota-derived uremic toxins, we performed untargeted LC-MS-based metabolomics on fecal samples from Sham, RIRI, and PN-CDs-treated mice. In comparison with the RIRI group, the PN-CDs group exhibited upregulation of 167 metabolites and downregulation of 62 metabolites **(Figure 9A)**. Hierarchical clustering analysis could show the difference of differentially expressed metabolites in different groups **(Figure 9B)**. We carried out GSEA to find the metabolic pathways that are enriched by different metabolites. Analysis indicates that the differentially expressed metabolites primarily participate in several metabolic pathways, with the tryptophan pathway being the most critical. PN-CDs downregulated the levels of tryptophan, indole, and indoleacetic acid within the tryptophan metabolic pathway **(Figure 9C)**. It indicates that PN-CDs may take effect through gut microbiota-tryptophan-indole axis. To prove this point, we analyzed quantitatively the metabolites related to the tryptophan metabolic pathway of the differential metabolites. Findings showed the RIRI group had a big rise in typical uremia toxins like p-cresyl sulfate **(Figure 9D)** and indoxyl sulfate **(Figure 9E)** when compared to the sham group. On the contrary, PN-CDs considerably cut down their levels. Additionally, we identified PN-CDs modulating other metabolites involved in the tryptophan-indole metabolic pathway, such as 6-hydroxyindoxyl sulfate **(Figure S10A)**, N-acetylserotonin sulfate **(Figure S10B)** and indole-3-acetamide **(Figure S10C)**.

To make a more correct study about PN-CD and the tryptophan-indole pathway, we used a quantitative metabolism pathway with LC-MS/MS technology. The PCA of metabolic profiles revealed distinct metabolic profiles for tryptophan metabolism in Sham, RIRI, and PN-CDs groups **(Figure 9F)**. The KEGG pathway analysis showed that the tryptophan metabolism pathway was significantly enriched **(Figure 9G)**. Tryptophan is first broken down to indole by gut microbial tryptophanase. Indole is subsequently absorbed and converted to the uremic toxin indoxyl sulfate in the liver. Statistical analysis on the key intermediates in this pathway showed a significant change; the Indoxyl sulfate was upregulated in RIRI but brought down to near physiological level by PN-CDs **(Figure 9H)**. Protective metabolites such as β-indole-3-acetic acid (IAA), indolylpropionic acid (IPA), and indole-3-lactic acid (ILA) showed no significant changes or decreased RIRI. However, these metabolites were highly upregulated by PN-CDs as well, in line with the untargeted metabolomics result **(Figures 9I-K)** in accordance with the untargeted metabolomics result. All these data together suggest that PN-CDs may program the branched indole metabolism of gut microbiota by suppressing the toxic branch (indole→indoxyl sulfate) and boosting the protective branch (tryptophan→IAA/IPA), and thus alleviating RIRI.

We carried out transcriptomic sequencing on kidney tissue from Illumina to research potential mechanisms. Clustering analysis of DEGs showed different transcriptional profiles between Sham, RIRI and PN-CDs groups **(Figure S10D)**. Compared with the RIRI group, the PN-CDs group had 604 upregulated and 697 downregulated genes **(Figure S10E)**. Venn diagram analysis showed 595 overlapped DEGs between RIRI vs. Sham and PN-CDs vs. RIRI comparisons **(Figure 9L).** Subsequent R-based enrichment analysis of these core genes-hose that are upregulated in RIRI vs. sham, but downregulated in PN-CDs vs. and vice versa, showed GO enrichment mostly in biological processes of metabolic regulation** (Figure S10F)**. And KEGG pathway analysis showed not only classic inflammatory signaling pathways, such as PPAR and MAPK but also tryptophan metabolism **(Figure 9M, Figure S10G-H)**. In order to verify the above-mentioned result from the transcriptomic data, the downstream gene expression of MAPK/ERK signaling pathway (MEK-1, MEK-2, ERK-1) has been quantified using qPCR method. From the results, it was found that PN-CDs are able to downregulate the MEK-1 mRNA, MEK-2 mRNA, and ERK-1 mRNA, as shown in **Figure S10 I-L**. These data indicate that PN-CDs can achieve their anti-inflammatory effects by regulating the MAPK/ERK signaling pathway.

Tryptophan-indole metabolites such as IAA and IPA are ligands for AHR, which is a cytoplasmic receptor and transcription factor that activates upon ligand binding to regulate pathophysiological processes by induction of target genes such as CYP1A1, CYP1A2, and CYP1B1^40^. To validate AHR pathway involvement, qPCR analysis demonstrated that RIRI significantly downregulated mRNA expression of AHR, CYP1B1, CYP1A1, and CYP1A2, whereas PN-CDs treatment restored their expression **(Figure 9N-O, Figure S10 M-N)**. Western blotting analysis further indicated that RIRI resulted in decreased levels of AHR protein expression and an elevation in the levels of phosphorylated extracellular regulated protein kinases (p-ERK). Conversely, PN-CDs restored AHR expression and suppressed p-ERK levels **(Figure 9P, Figure S11 A-C)**, suggesting the involvement of the AHR-ERK signaling axis.

Collectively, integrated untargeted metabolomics, targeted tryptophan metabolomics, and transcriptomic analyses consistently indicate that PN-CDs exert renal protection through modulation of tryptophan metabolism. Mechanistically, PN-CDs may activate AHR signaling while inhibiting ERK phosphorylation, thereby suppressing inflammatory cytokine expression and mitigating RIRI.

### 2.9 *In vitro* and *in vivo* bio-safety of PN-CDs

**Figure 10:**
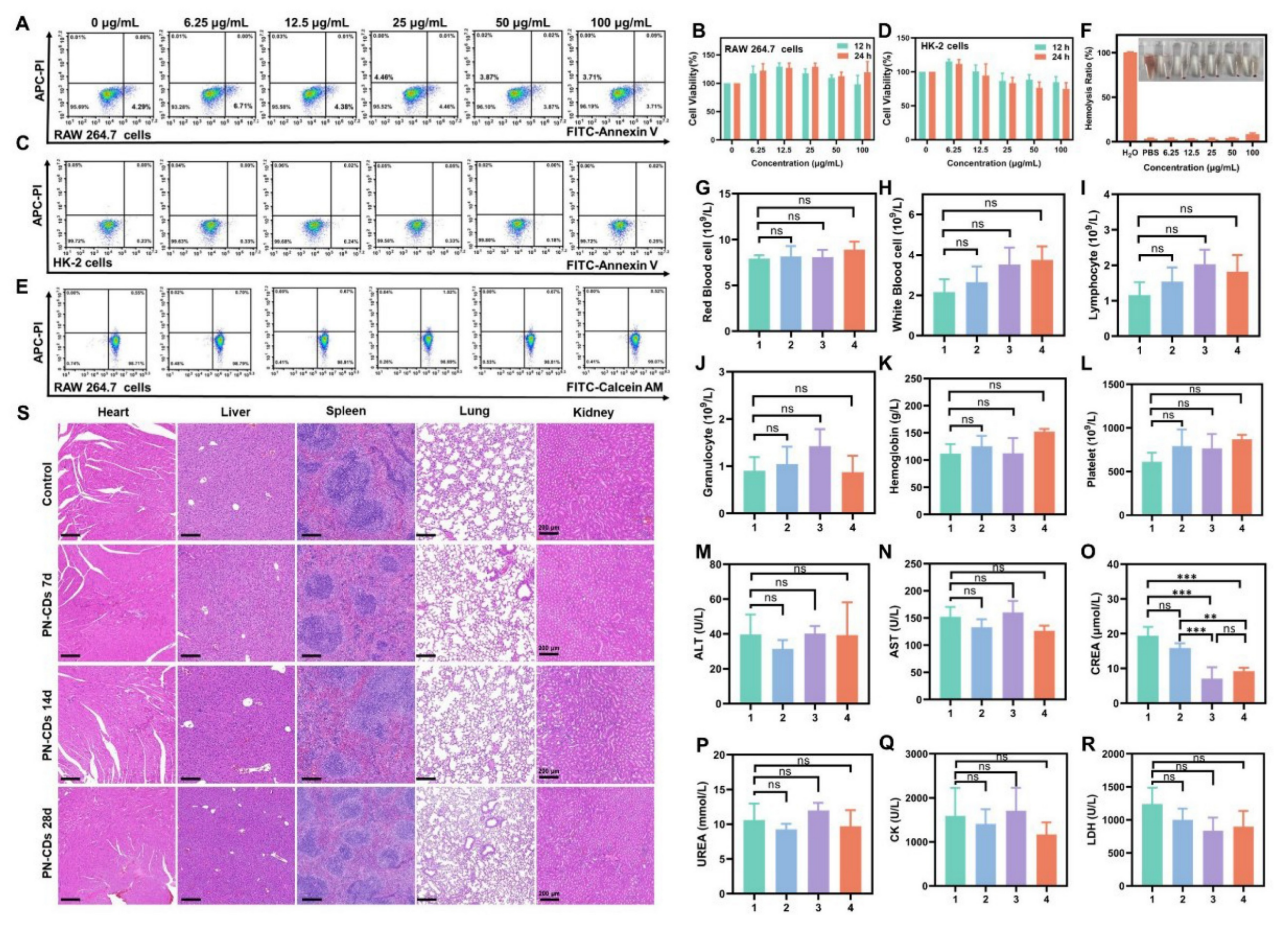
**Biosafety evaluation of PN-CDs *in vitro* and *in vivo*.** (A) Flow cytometry analysis of apoptosis in RAW 264.7 cells treated with PN-CDs (0-100 μg/mL) for 24 h, stained with FITC-Annexin V/APC-PI. (B) CCK-8 assay for viability of RAW 264.7 cells after 12 h and 24 h incubation with PN-CDs. (C) Flow cytometry analysis of apoptosis in HK-2 cells treated with PN-CDs (0-100 μg/mL, 24 h), stained with FITC-Annexin V/APC-PI. (D) CCK-8 assay for viability of HK-2 cells after 12 h and 24 h incubation with PN-CDs. (E) Calcein-AM/PI live-dead staining of RAW 264.7 cells treated with PN-CDs (0-100 μg/mL, 24 h), analyzed by flow cytometry. (F) Hemolysis assay: Red blood cells were incubated with PN-CDs (0-100 μg/mL) at 37°C for 4 h, and the hemolysis rate was quantified. (G-L) Hematological profiling of mice after intraperitoneal injection of PN-CDs (20 mg/kg) for 7 days (group 2), 14 days (group 3) or 28 days (groups 4): (G) red blood cells, (H) white blood cells, (I) lymphocytes, (J) granulocytes, (K) hemoglobin, (L) platelets. (M-R) Serum biochemical analysis: (M) alanine transaminase (ALT), (N) aspartate transaminase (AST), (O) creatinine, (P) blood urea nitrogen (BUN), (Q) creatine kinase (CK), (R) lactate dehydrogenase (LDH).1: Control; 2: PN-CDs for 7 days; 3: PN-CDs for 14 days; 4: PN-CDs for 28 days. (S) H&E staining of heart, liver, spleen, lung, and kidney tissues from control mice, and those treated with PN-CDs for 7 days, 14 days or 28 days. Data are presented as mean ± SEM; ns, not significant, *P < 0.05, **P < 0.01, **P < 0.001.

Finally, we assessed the biosafety of PN-CDs to lay a foundation for subsequent clinical translation. Flow cytometry and CCK-8 assays revealed that PN-CDs in concentrations between 0 and 100 μg/mL did not significantly affect apoptosis or viability of RAW 264.7 cells **(Figure 10A, B)** and HK-2 cells** (Figure 10C, D)** after 12 and 24 hours of incubation. Calcein-AM/PI staining confirmed 99.07% viability in RAW 264.7 cells at a concentration of 100 μg/mL after 24 hours, indicating minimal cytotoxicity **(Figure 10E)**. The hemolysis assay indicated an 8.5% hemolysis rate after incubating red blood cells with 100 μg/mL PN-CDs at 37°C for 4 hours **(Figure 10F)**.

At the animal level, we assessed *in vivo* biosafety by intraperitoneally injecting PN-CDs at a daily dose of 20 mg/kg throughout 7, 14, and 28 continuous days. The blood samples and organs after being treated were collected for the exam. In blood routine analysis, there was no significant effect of PN-CDs on RBC **(Figure 10G)**, WBC** (Figure 10H)**, Lymphocytes** (Figure 10I),** Granulocytes** (Figure 10J)**, Hemoglobin **(Figure 10K)**, and Platelets **(Figure 10L)**. Serum biochemical analysis to examine liver, kidney and heart function did not show any major alterations of ALT **(Figure 10M)** and AST **(Figure 10N)** after 7, 14, and 28 days of treatment with PN-CDs. Additionally, it was also injected for 14 days and 28 days for PN-CDs to reduce the level of serum creatinine **(Figure 10O)** without influencing the BUN level **(Figure 10P)**. Likewise, the continuous injection of PN-CDs did not change CK and LDH levels **(Figure 10Q-R)**. The results show that the PN-CDs are compatible and safe in both lab and living organisms.

## 3. Discussion

In this study, PN-CDs herbzymes were obtained using *Panax notoginseng* as a precursor. We have revealed that PN-CDs display antioxidant enzyme activity and free radical-eliminating abilities. Anti-inflammation and oxidation effects. *In vivo*, PN-CDs could be accumulated in the injured kidneys and significantly relieved RIRI. In multi-omics mechanistic analysis, it is found that PN-CDs regulate gut microbiota homeostasis and tryptophan metabolism, reduce the levels of renal toxin indoxyl sulfate, and subsequently activate the AHR pathway while inhibiting the inflammatory signaling pathway. These effects together reduce inflammation and lipid peroxidation damage in RIRI.

Nanozymes, catalytically active nanomaterials, can be classified by their synthetic precursors, including transition metals, metal oxides, carbon-based structures, metal-organic frameworks, and single-atom catalysts^41^. This study successfully created *Panax notoginseng* CDs nanozymes using the traditional Chinese herb *Panax notoginseng*. These nanozymes offer significant advantages over conventional ones. Traditional nanozymes often require non-renewable materials, harsh conditions, and complex procedures. For example, Gao *et al*. needed strong oxidizing agents and a lengthy process to synthesize inorganic CDs nanozymes, risking unreacted residues^42^. CeO₂ is commonly produced using template-assisted, sol-gel, or coprecipitation methods. Coprecipitation often results in low-purity products with inconsistent particle sizes and shapes. Template-based methods have challenges with template removal and strict processing requirements, while sol-gel techniques are costly due to expensive metal alcoholates^43^. Mn₃O₄ is typically made through chemical precipitation, pyrolysis, or hydro/solvothermal methods, followed by calcination, complicating morphology and size control and involving organic solvents and complex procedures. Pyrolysis can cause grain sintering and reduced surface area due to high temperatures^44^. In contrast, PN-CDs are created using simple hydrothermal methods without extra organic materials, under mild conditions. Natural groups like hydroxyl and carboxyl in *Panax notoginseng* form enzyme-like active sites, avoiding toxic reducing agents and cutting environmental risks and costs. Traditional nanozymes are difficult to mass-produce due to reliance on non-renewable materials, complex processes, and strict purification needs. Inorganic nanozymes like CeO₂ and Mn₃O₄ also face high material costs and scaling challenges. Conversely, herbal resources are abundant and easily accessible, and the simple synthesis of PN-CDs without complex equipment supports large-scale production.

PN-CDs are taken up by resting macrophages as well as M1-type macrophages, as seen in Figure 4 and Figure S2. It is because the internalization capacity of these materials exists because the mononuclear phagocyte (MPS) has a macrophage population that can already identify and break down foreign bodies such as nanomaterials. Nevertheless, *in vivo* imaging results in Figure 6 demonstrate that *in vitro* internalization does not necessarily result in rapid *in vivo* clearance of the particles by the MPS. In particular, Figure 6 shows that PN-CDs tend to gather in the kidneys rather than the liver and spleen. In addition to quantitative fluorescence analysis, the results also indicate that 12 hours after intraperitoneal injection of the fluorescent probe, the fluorescence intensity in the kidneys was greater than that in the liver, being approximately 3.17 times higher (Figure 6H-I). This shows that PN-CDs are able to target the kidneys while not being cleared nonspecifically by the MPS. This phenomenon might be caused by the unique physicochemical characteristics of PN-CDs: PN-CDs are about 2nm in size, which can decrease the non-specific adsorption of plasma proteins and weaken the recognition efficiency of MPS^45^. Concurrently, PN-CDs themselves possess anti-inflammatory properties, capable of both inhibiting macrophage activation (Figure 5E-G) and to some extent reducing the recognition capacity of the mononuclear phagocyte system (MPS). Which suggests that PN-CDS accumulates in pathologic macrophages for a therapeutic benefit and is not quickly captured and degraded by MPS cells that are quiescent in the liver and spleen. In summary, though PN-CDs can be internalized by RAW 264.7 macrophages *in vitro*, the *in vivo* imaging results, as well as the unique physicochemical characteristics, together indicate that PN-CDs could achieve prolonged systemic circulation and accumulate in the target organs without rapid clearance by MPS. The balance of "targeted interaction with MPS cells" and "MPS evasion (for long circulation purposes)" is important for the efficacy of MPS cells in RIRI.

Herbzymes' catalytic mechanism is analogous to that of CDs nanozymes, pH, temperature, reducing agent, surface groups, and functionalization degree. Through surface modification and theoretical calculations, Gao *et al*. demonstrated that the SOD-like activity of carbon nanotubes depends on the binding of hydroxyl and carboxyl groups with superoxide anions, as well as electron transfer involving the carbonyl group conjugated with the π system. Hydroxyl and carboxyl groups in CDs bind •O₂⁻, while carbonyl groups oxidize •O₂⁻ to generate oxygen and reduced CDs. These reduced CDs are subsequently reoxidized by another •O₂⁻, resulting in H₂O₂ production^46^. Deng *et al*. utilized hydrothermal and carbonization techniques to synthesize honeysuckle-derived CDs exhibiting SOD enzymatic activity. Through comparison analysis, superficial group modification, and theory calculations, they discovered that the SOD-like activity of carbon nanotubes primarily stems from the nitrogen-based stabilisation of the •O₂⁻ intermediate and its synergistic effects. Additionally, CDs synthesized by hydrothermal method (HyCDs) with higher surface functional group density and polymer chain density exhibit higher SOD-like enzymatic activity^24^. In this study, the synthesized PN-CDs were also found to possess SOD enzymatic activity, effectively scavenging ROS at the *in vitro* and cellular levels. FT-IR and XPS investigations revealed the existence of hydroxyl, amino, and carbonyl groups present on the surface of PN-CDs (Figure 2G-L). DFT calculations indicate that the carbonyl groups on the PN-CDs surface, which are conjugated with the π system, act as active sites. These sites facilitate charge transfer and spontaneous energy changes during catalytic cycles, enhancing SOD-like activity and •OH scavenging, thus promoting enzymatic reactions and intrinsic antioxidant effects.

Kidney disease patients experience gut microbiota dysbiosis, with the gut microbiota influencing kidney injury through various pathways, including the modulation of local and systemic immunity, as described by the gut-kidney axis theory^47^. Ruan *et al*. demonstrated that oral administration of black phosphorus quantum dots causes gut microbiota dysbiosis, reduces the abundance of *Lactobacillus* and the content of acetic acid, and induces renal T cell activation, inflammation, and fibrosis in mice. Notably, supplementation with *Lactobacillus* and its metabolites can alleviate renal inflammation and fibrosis^48^. These findings suggest that nanoparticles can mediate renal injury through the gut microbiota-metabolite-kidney axis. In this study, PN-CDs were found to alter the species and abundance of gut microbiota in RIRI model mice, reducing the levels of harmful bacteria such as *Escherichia-Shigella*, *Erysipelatoclostridium*, and *Muribaculum*, while increasing the abundance of *Rikenellaceae_RC9_gut_group* (Figure 8). *Escherichia-Shigella* are common intestinal pathogens that can damage renal endothelial cells by directly releasing Shiga toxins, affect the accumulation of renal toxins, and indirectly exacerbate kidney injury by influencing oxidative stress and inflammatory responses^49^, ^50^. However, studies on *Erysipelatoclostridium*, *Muribaculum*, and *Rikenellaceae_RC9_gut_group* in kidney injury models are limited. From the findings of this research, we propose that *Erysipelatoclostridium* and *Muribaculum* may contribute to kidney damage by increasing renal toxins and activating inflammation and oxidative stress signaling pathways. *Rikenellaceae_RC9_gut_group* generates short-chain fatty acids like butyric acid, known for their anti-inflammatory and antioxidant properties, potentially safeguarding the kidney by reducing renal inflammation and oxidative stress^51^, ^52^.

Both metabolomic and transcriptomic results in this study confirmed that the mechanism by which PN-CDs alleviate kidney injury is associated with tryptophan-indole metabolism-related signaling pathways. PN-CDs reduce indoxyl sulfate levels and increase IAA, IPA, and ILA (Figure 9). Indole compounds, mainly produced by gut microbial metabolism, include IAA, IPA, ILA, indole-3-aldehyde (I3A), and indoxyl sulfate, among others, and these compounds exert dual effects in kidney injury. Indoxyl sulfate is a known uremic toxin that can induce oxidative stress and inflammatory responses, exacerbating kidney injury; in contrast, indole-3-aldehyde, together with indole-3-acetic acid exhibit antioxidant and anti-inflammatory effects, which can alleviate kidney injury^53^-^55^. Furthermore, IAA, IPA, and ILA can act as ligands for the AHR receptor, activating AHR, which in turn induces the activation of subsequent genes like cytochrome P450 enzymes and cytokines, thereby regulating intestinal homeostasis, immune responses, and metabolism^56^. In this experiment, qPCR and Western blotting results verified that PN-CDs upregulate the expression levels of AHR at both mRNA and protein stages and its downstream gene CYP1B1, consistent with previous research conclusions.

In a word, the PN-CDs herbzymes synthesized in this paper have antioxidant enzyme activity, which is related to the carbonyl groups present on the surface of CDs. PN-CDs alleviate RIRI by modulating the gut microbiota-tryptophan-indole-aromatase pathway, thereby exerting anti-inflammatory effects and mitigating oxidative stress.

## 4. Conclusion

In the current study, we constructed the PN-CDs herbzymes originated from the Chinese herb *Panax notoginseng* with endogenous antioxidant enzymatic activity and anti-inflammatory activity that could alleviate RIRI. Our findings together show that the PN-CDs herbzymes extended the systemic circulation time, improved the kidney targeting, and enhanced mitochondrial delivery. Lots of in test tube and in the living, body show they are good at cleaning up harmful stuff in the blood and stopping swelling, so they help kidneys not get hurt as much. Mechanistically, PN-CDs confer renoprotective effects through a multifaceted pathway: they regulate gut microbiota homeostasis, reduce the renal toxin indoxyl sulfate, increase beneficial indole metabolites, activate the AHR, and attenuate ERK inflammatory signaling cascade. PN-CDs are notable for showing excellent biocompatibility and biosafety on both the lab and live models which make them a possible therapeutic for RIRI. Overall, our work provides a promising nanomedicine strategy to alleviate renal injury through effectively modulating oxidative stress and gut microbiota-tryptophan-AHR metabolic axis. It can create a new idea about how to treat RIRI but it would also increase the range of possibilities for Chinese herbs and it would be good to increase nanotechnology-assisted herbal treatment drug.

## 5. Materials and Methods

***Materials*:** *Panax notoginseng* was sourced from Xi'an Kailai Bioengineering Co., Ltd**.** Sulfo Cy5.5 NH2 was sourced from Xi'an Ruixi Biotechnology Co., Ltd. The ABTS assay kit, Anti fluorescence quenching sealing solution (with DAPI), Mito-Tracker Green, the JC-1 detection kit, along with the RNAeasy™ Animal RNA Isolation Kit with Spin Column, and dihydroethidium (DHE) probe were purchased from Beyotime (Shanghai, China). The DCFH probe and TMB were obtained from Solarbio (Beijing, China). Ltd. The SOD detection kit (WST method) was obtained from Dojindo (Shanghai, China). Cellulose dialysis bags with a 1000 Dalton cutoff were provided by Shanghai Yuanye Bio-Technology Co., Ltd. Suzhou Youyi Randy Biotechnology Co., Ltd provided the Cell Counting Kit-8 (CCK-8) and FITC Annexin V/PI Apoptosis Kit. (uelandy). The Lipid Peroxidation MDA Assay Kit, along with the GSH and GSSG Assay Kit and mouse TNF-α ELISA kit, were sourced from ABclonal Technology Co., Ltd.

***Preparation of PN-CDs:*** The lumpy *Panax notoginseng* was ground into powder. A homogeneous mixture was prepared by thoroughly stirring 100 mg of* Panax notoginseng* powder in 10 milliliters of ultrapure water for 30 minutes. The suspension was placed in a 100 mL Teflon-lined autoclave and heated at 180 °C for 12 hours. After cooling to room temperature, the mixture was centrifuged at 10,000 rpm for 20 minutes. The supernatant was filtered through a 0.22 µm polyethersulfone membrane after discarding the pellet. The solution was purified by dialysis against ultrapure water using 1000 Da dialysis bags for 4 days, followed by freeze-drying to obtain the final product.

***Characterization of PN-CDs:*** PN-CDs were morphologically characterized using transmission electron microscopy (TEM) and atomic force microscopy (AFM). The surface groups and structural characteristics of PN-CDs were examined using various spectroscopic and analytical techniques, including UV-Vis spectroscopy (SHIMADZU UV-2700), fluorescence spectroscopy (HITACHI F-4700), Fourier transform infrared spectroscopy (FT-IR, Nicolet 6700, Thermo Scientific), Raman spectroscopy, X-ray diffraction (XRD), proton nuclear magnetic resonance spectroscopy (¹H NMR), and X-ray photoelectron spectroscopy (XPS). Raman, XRD, ¹H NMR, and XPS analyses were conducted at Beijing Zhongkebaice Technology Service Company, Ltd.

***Synthesize PN-CDs-Cy5.5:*** To activate the carboxyl groups, 100 μL of EDC solution (10 mg/mL) was combined with 200 μL of NHS solution (10 mg/mL) and incubated at 25°C for 30 minutes. 1 mL of PN-CDs solution (5 mg/mL) was added to the EDC/NHS-activated mixture and stirred in the dark at room temperature for 1 hour to promote amide bond formation. Subsequently, 2 mL of Cy5.5 solution (0.25 mg/mL) was added to the reaction system and stirred continuously at room temperature for 24 hours in the dark. The final product, PN-CDs-Cy5.5, was purified by dialysis against ultrapure water using a 1000 Da membrane for 24 hours, resulting in a red-fluorescent conjugate.

***Cell culture:*** RAW 264.7 and HK-2 cells were kept at 37 °C in an environment with 5% CO_2_. in DMEM and F12/DMEM media, respectively, both supplemented with 10% fetal bovine serum, 100 U/mL penicillin, and streptomycin.

**ROS Scavenging ability of PN-CDs *in vitro*:** *•ABTS^+^ free radical scavenging activity:* The ability of PN-CDs to scavenge •ABTS^+^ free radicals was evaluated by creating an •ABTS^+^ stock solution (7.4 mmol/L, 0.4 mL) and a K₂S₂O₈ stock solution (2.6 mmol/L, 1.43 mL). •ABTS^+^ radical scavenging activity was assessed for PN-CDs by preparing an •ABTS^+^ stock solution (7.4 mmol/L, 0.4 mL) and a K₂S₂O₈ stock solution (2.6 mmol/L, 1.43 mL). The solution was stirred and let sit for a full 12 hour period in darkness. Before using, it was diluted 50 times with anhydrous ethanol. Scavenging percentage of •ABTS^+^ radicals by PN-CDs was obtained from a calibration curve made with UV-Vis absorbance measurements. In the experiment, 10 uL of different concentrations of PN-CDs were added into •ABTS^+^, the UV-Vis absorbance was detected every 60s for a total of 600s.

*•O_2_^-^ scavenging activity:* PN-CDs' •O₂⁻ scavenging efficacy was tested with NBT as a probe. A solution system was prepared with riboflavin (6.67 μM), L-methionine (4.33 mM), NBT (25 μM), and varying concentrations of PN-CDs, everything was dissolved in a 0.01 M PBS buffer with a pH of 7.4. Afterward, the solution was exposed to light from a 30 W illuminator tube. The solution's absorbance at 560 nm was measured every 10 seconds during illumination. Finally, the reaction kinetics were deduced from the obtained absorbance data.

*SOD enzyme activity detection:* SOD enzyme activity was assessed using a WST-8 SOD assay kit (Dojindo) according to the manufacturer's protocol. A 200 μL reaction mixture was assembled in a 96-well plate, consisting of 100 μL WST-8 solution, 20 μL enzyme solution, and 80 μL of either varying concentrations of PN-CDs or a blank control (0.01 M PBS buffer). A positive control used the SOD standard from the kit. After mixing, the plate was kept in the dark at 37 °C for 30 minutes. Absorbance at 450 nm was measured using a microplate reader. SOD activity (U/mL) was calculated with the formula: [(A₀-Aₛ) /A₀] × 2 × dilution ratio, where A₀ represents the blank control absorbance and Aₛ denotes the sample absorbance. Each sample was tested in triplicate for accuracy.

*•OH scavenging activity:* The •OH scavenging activity of PN-CDs was investigated by constructing a •OH generation system via the Fenton reaction of Fe²⁺ and H₂O₂, with TMB serving as a color developer to reflect the •OH content. The prepared solution system included Fe²⁺ (10 mM), H₂O₂ (100 mM), TMB (10 mM), and PN-CDs at various concentrations. Throughout the reaction, the absorbance of the solution system at 652 nm was detected every 10 seconds. Based on these absorbance values, the reaction kinetics of the •OH scavenging process by PN-CDs were calculated.

***Cellular internalization and mitochondrial co-Localization of PN-CDs-cy5.5:*** The internalization and mitochondrial co-localization of PN-CDs-Cy5.5 were evaluated in RAW 264.7 and HK-2 cells. RAW 264.7 and HK-2 cells cultured on glass slides were exposed to 20 μg/mL PN-CDs-Cy5.5 for varying durations of 2, 4, 6, and 8 hours. Post-incubation, cells underwent 3-5 PBS washes to eliminate non-internalized PN-CDs-Cy5.5. For fluorescent staining, cell nuclei were labeled with DAPI, and the cytoskeleton was stained with Phalloidin. Fluorescence microscopy was employed to observe and image the stained cells. To assess mitochondrial localization of PN-CDs, RAW 264.7 and HK-2 cells on glass slides were treated with 20 μg/mL PN-CDs-Cy5.5 for 5 hours. Nuclei and mitochondria were stained using DAPI and Mito-tracker, respectively. The colocalisation of PN-CDs-Cy5.5 with mitochondria was analysed by means of ImageJ software.

***Intracellular anti-inflammatory and antioxidant effects of PN-CDs:*** The anti-inflammatory and antioxidant properties of PN-CDs were assessed using RAW 264.7 cells, which were exposed to LPS or H₂O₂ to create models of inflammation and oxidative damage, respectively. The anti-inflammatory effects of PN-CDs were assessed by co-incubating RAW 264.7 cells with PN-CDs (1 μg/mL, 5 μg/mL) and LPS (100 ng/mL) for 12 hours, subsequently, intracellular ROS was detected using DCFH-DA as a fluorescent marker. Fluorescence microscopy and flow cytometry were used to evaluate the capacity of PN-CDs to lower ROS levels. mRNA expression levels of inflammatory factors were evaluated using qPCR across different treatment groups. To assess the antioxidant capacity of PN-CDs, RAW 264.7 cells were exposed to PN-CDs at concentrations of 1 μg/mL and 5 μg/mL, along with 100 μM H₂O₂, for 12 hours. Intracellular total ROS and superoxide anion radicals (•O₂⁻) were measured using DCFH and DHE fluorescent probes, respectively. The scavenging effects of PN-CDs on total ROS and •O₂⁻ generated by oxidative damage were analyzed via fluorescence microscopy and flow cytometry.

***Establishment of the Cell Hypoxia-Reoxygenation Model:*** HK-2 cells were planted and grown in F12/DMEM complete medium until they reached about 70% confluence. During hypoxia induction, the medium was switched to serum-free, low-glucose DMEM pre-equilibrated under hypoxic conditions. The cells were first treated with PN-CDs at the start of the H/R model establishment and this treatment was maintained until the model was completed. Cells were incubated in a hypoxic workstation (1% O₂, 5% CO₂, 94% N₂) for 24 hours, followed by reoxygenation in fresh F12/DMEM medium for 12 hours. The anti-apoptotic activity of PN-CDs in the H/R model of HK-2 cells was assessed using Calcein-AM/PI staining, Annexin V/PI apoptosis detection, and CCK-8 assays. Mitochondrial protection and ROS scavenging capabilities were evaluated using the JC-1 mitochondrial staining kit and DCFH-DA fluorescent probe. The redox state was assessed using a Lipid Peroxidation MDA Assay Kit and a GSH/GSSG Assay Kit. The mRNA expression levels of kidney injury biomarkers KIM-1 and NGAL were evaluated through qPCR analysis.

***Animals:*** Male C57BL/6 mice, aged eight weeks, were obtained from the Medical Experimental Animal Center at Xi'an Jiaotong University in Shaanxi, China. They were housed at a temperature of 22-25 °C with a humidity level of 65±5%, under a 12-hour light-dark cycle, and had regular access to drinking water. The animal experiments were conducted in accordance with Xi'an Jiaotong University's Guidelines for the Care and Use of Laboratory Animals and were approved by the Animal Ethics Committee.

***Establishment of mouse renal ischemia-reperfusion injury model:*** Bilateral renal pedicles of mice were clamped using vascular clips for 28 minutes, followed by release of the clips. Subsequently, the muscular layer and skin were sutured. After the mice regained consciousness, they were intraperitoneally injected with PN-CDs at doses of 1 mg/kg and 5 mg/kg, as well as saline in the control group, according to different treatment protocols. 24 h post-injection, fecal samples and kidney tissues were collected from the mice, which were then utilized for multi-omics analysis, H&E, PAS, immunohistochemical staining, and other subsequent experiments.

***In vivo imaging:*** Mice in both the healthy control and IRI groups received an intraperitoneal injection of PN-CDs-Cy5.5 at a dose of 5 mg/kg. *In vivo* imaging of the entire abdomen and various organs of mice was conducted at 0, 0.5, 1, 2, 4, 8, 12, and 24 hours post-injection using the VISQUE® *In Vivo* imaging system to monitor the distribution and kinetics of PN-CDs-Cy5.5.

***Muti-omics processing of mouse fecal and kidney tissue:*** 16S rRNA, untargeted metabolomics, and eukaryotic transcriptomics were performed by Novogene Co., Ltd. (Beijing, China), and the analyses were conducted using the NovoMagic cloud platform. The tryptophan-targeted metabolomics was completed by Metware Biotechnology Co., Ltd. (Wuhan, China).

***16S rRN****A*: Fecal samples from mice in different treatment groups were collected for genomic DNA extraction and sample detection. Bacterial diversity was assessed using 16S rRNA V4 region primers, 515F and 806R. The PCR products were amplified, mixed, and purified by agarose gel electrophoresis. Following the library qualification, PE250 sequencing was conducted using NovaSeq6000, followed by bioinformatics analysis.

***Untargeted metabolomics*:** Untargeted metabolomics was performed. The metabolites of the mice feces were extracted and then identified and detected by LC-MS, and finally analyzed.

***Eukaryotic transcriptomics:*** For eukaryotic transcriptomics, RNA was extracted from tissues/cells with common methods, and then RNA quality was controlled with Agilent 2100 bioanalyzer. Libraries were made by the NEB general method or the NEB strand-specific method. Following library construction, the library was quantified using a Qubit 2.0 fluorometer, subsequently diluted to a concentration of 1.5 ng/μL. The insert size of the library was detected by the Agilent 2100 bioanalyzer. After confirming that the expected insert size was correct, we used qPCR to quantify the effective library, with the effective library greater than 1.5 nM and it is suitable for use.

***Tryptophan-targeted metabolomics****:* Metabolite extraction was performed on kidney tissues from mice across various treatment groups for tryptophan-targeted metabolomics. Subsequently, metabolites were identified using liquid chromatography-tandem mass spectrometry (LC-MS/MS) was used to detect the metabolites. The qualitative analysis of mass spectrometry data was performed based on the MWDB (Metware Database) constructed using standard products. Finally, data analysis of metabolites was conducted.

***Western blotting:*** Kidney tissue samples were homogenized on ice for 30 minutes in RIPA lysis buffer (Beyotime) mixed with 1× protease/phosphatase inhibitor cocktail to extract total proteins. Using 10% polyacrylamide gel electrophoresis (PAGE), proteins were isolated and then moved to 0.22 μm polyvinylidene fluoride (PVDF) membranes. The membranes were treated with a 5% non-fat milk solution to block non-specific binding, targeting AHR (Abways, CY3431), ERK (Servicebio, GB11560), and phosphorylated ERK (P-ERK) (Servicebio, GB11004). Following a rinse with TBST buffer containing 2% Tween-20 to eliminate unbound primary antibodies, the membranes were incubated at room temperature for 2 hours with HRP-conjugated secondary antibodies (Servicebio, GB23303). The ChemiScope 6100 imaging system was used to visualize and capture the chemiluminescent signals of the protein bands.

***Biosafety evaluation:*** For the biosafety evaluation, experiments were conducted at both the cellular and *in vivo* levels. At the cellular level, RAW 264.7 and HK-2 cells were exposed to varying concentrations of PN-CDs for either 12 or 24 hours. Subsequently, an Annexin V/PI apoptosis kit, and the CCK-8 kit were employed to verify the impact of PN-CDs on cell viability. In the *in vivo* experiments, mice were intraperitoneally injected with normal saline and 20 mg/kg PN-CDs continuously for 7 days, 14 days, and 28 days respectively. Later, the mice were euthanized. Blood samples were collected for standard and biochemical tests, and vital organs were gathered for H&E staining to assess pathological conditions.

***Statistical analysis:*** Statistical analysis involved using GraphPad Prism 9.0 software for data evaluation. The results are shown as mean ± SD from three independent experiments. An unpaired two-tailed t-test was used to analyze differences between the two groups. A one-way ANOVA was also conducted, followed by Tukey's HSD test for post hoc analysis. Statistical significance denoted as * *p* < 0.05, ** *p* < 0.01, and *** *p* < 0.001.

## Supplementary Material

Supplementary figures.

## Abbreviations

RIRI: Renal ischemia-reperfusion injury
ROS: Reactive oxygen species
LPS: Lipopolysaccharide
AHR: aryl hydrocarbon receptor
NGAL: neutrophil gelatinase-associated lipocalin
KIM-1: kidney injury molecule-1
CDs: Carbon dots
PN-CDs: *Panax notoginseng* CD nanozymes
