# Supplementary material for: Panax notoginseng-Derived Carbon Dots Herbzymes Ameliorate Renal Ischemia-Reperfusion Injury via Anti-Inflammation, Antioxidation and Gut-Kidney Axis Regulation
Source: Theranostics. 2026 Jan 14;16(8):4019–41. doi: 10.7150/thno.126643 (PMC12905796; doi:10.7150/thno.126643)
Supplement: Supplementary file 1 — Supplementary figures. [file thnov16p4019s1.pdf]

Panax notoginseng-Derived Carbon Dots Herbzymes Ameliorate Renal Ischemia-Reperfusion Injury via Anti-Inflammation, Antioxidation and Gut-Kidney Axis Regulation

Mei Yang[1,3#], Zhenting Zhao[2,3#], Zhichao Deng[4], Shirui Sun[2,3], Yingcong Guo[2,3], Zepeng Li[2,3], Bingxuan Zheng[2,3], Chengbo Zhang[1,2], Jianhui Li[5], Hongbao Li[4], Mingzhen Zhang[4], Hua Wang[1,3], Kun Ye[6*], Wujun Xue[2,3*], Chenguang Ding[1,2,3*]

Affiliations:

1. Department of Organ Procurement and Allocation, the First Affiliated Hospital of Xi'an Jiaotong University, Xi’an, Shaanxi, 710061, China

2. Department of Kidney Transplantation, the First Affiliated Hospital of Xi’an Jiaotong University, Xi’an, Shaanxi, 710061, China

3. Institute of Organ Transplantation, Xi’an Jiaotong University, Xi’an, Shaanxi, 710061, China

4. School of Basic Medical Sciences, Xi’an Jiaotong University, Xi’an, Shaanxi, 710061, China

5. Department of Hepatobiliary and Pancreatic Surgery, Key Laboratory of Artificial Organs and Computational Medicine in Zhejiang Province, Shulan（Hangzhou）Hospital, Hangzhou, Zhejiang, 310022, China

6. Department of Nephrology, Guangxi Clinical Research Center for Chronic Kidney Disease, People's Hospital of Guangxi Zhuang Autonomous Region, Nanning, Guangxi, 530021, China

[#] These authors contributed equally to this work.

* Authors for correspondence

Kun Ye (ORCID: 0009-0006-8209-1090)
Email address: yk@gxams.org.cn

Wujun Xue (ORCID: 0000-0002-2833-7786)
Email address: xwujun126@xjtu.edu.cn

Chenguang Ding (ORCID: 0000-0001-9306-9709)

Email address: doctor_ding@xjtu.edu.cn

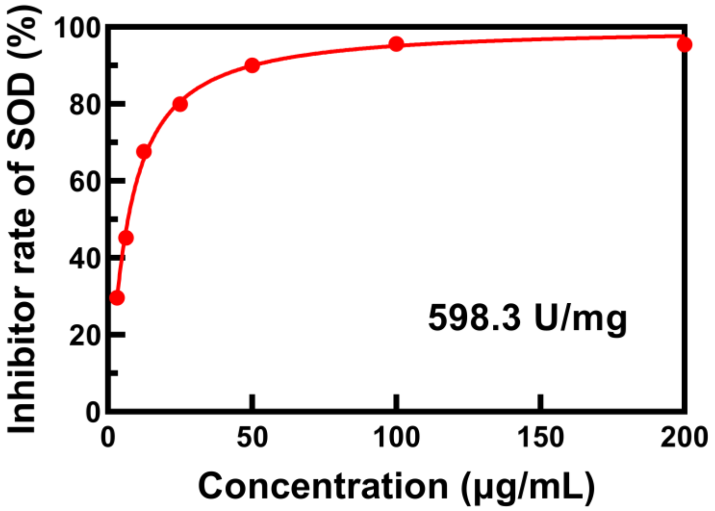

Figure S1. SOD-like activities of PN-CDs.

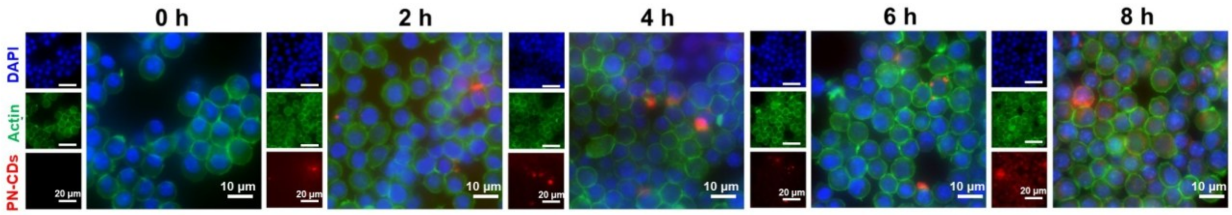

Figure S2. Fluorescence images showing the time-dependent cellular uptake of PN-CDs in naive RAW 264.7 cells. Cells were stained with DAPI (blue, nuclei) and Actin (green, cytoplasm); PN-CDs were labeled with Cy5.5 (red).

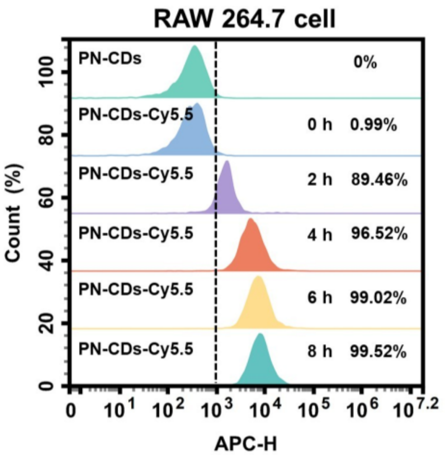

Figure S3. Flow cytometry quantification of PN-CDs-Cy5.5 uptake efficiency in naive RAW 264.7 cells at different time points (0, 2, 4, 6, 8 h).

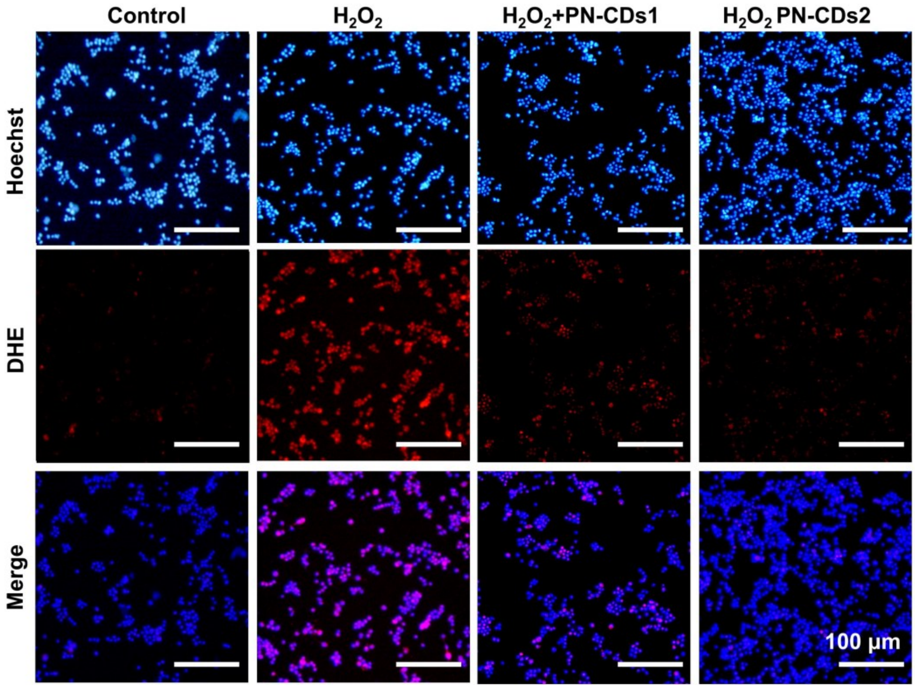

Figure S4. Confocal fluorescence images showing intracellular $O_2\bullet^-$ levels (red, DHE) and nuclear staining (blue, Hoechst) in RAW 264.7 cells treated with control, H_2O_2, H_2O_2 + PN-CDs1, or H_2O_2 + PN-CDs2.

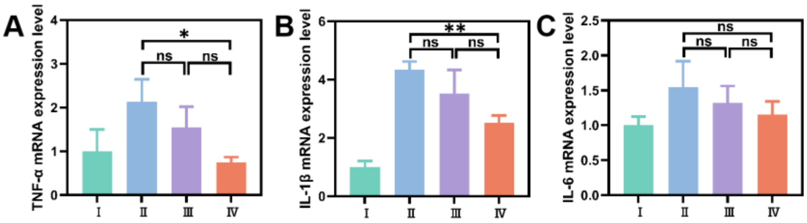

Figure S5. qPCR analysis of TNF-α (A), IL-6 (B) and IL-1β(C) mRNA expression in LPS induced HK-2 cell model. Data are presented as mean ± SD. Statistical significance: *p < 0.0, **p < 0.01 vs. LPS group; ns, not significant. I: Control; II: LPS; III: LPS + PN-CDs1; IV: LPS+ PN-CDs2.

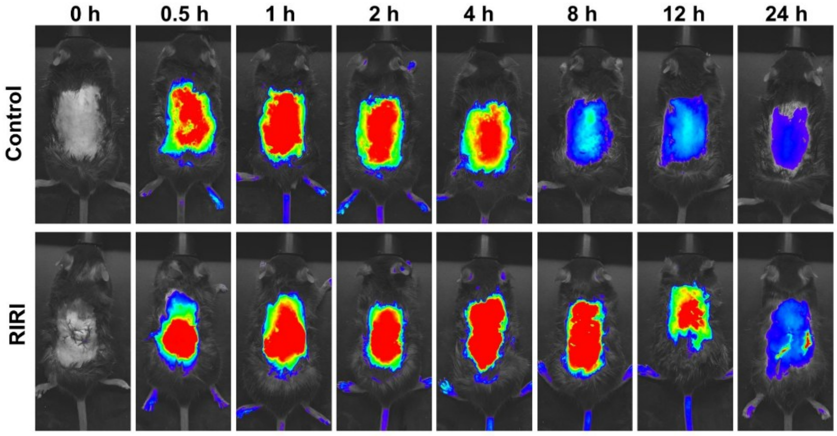

Figure S6. Whole-body fluorescence imaging of PN-CDs-Cy5.5 in control and RIRI model mice at 0 h, 0.5 h, 1 h, 2 h, 4 h, 8 h, 12 h, and 24 h post-intraperitoneal injection.

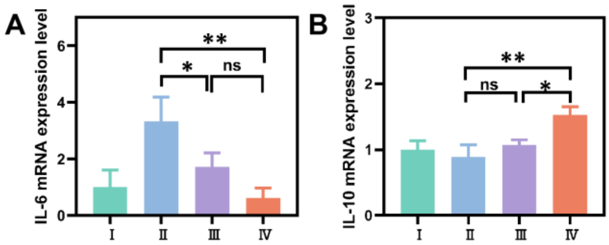

Figure S7. qPCR analysis of IL-6 and IL-1β mRNA expression levels in renal tissues following PN-CDs treatment for RIRI. Data are mean ± SEM; *p < 0.05, **p < 0.01 vs. RIRI group. I: Sham; II: RIRI; III: RIRI + PN-CDs1; IV: RIRI + PN-CDs2.

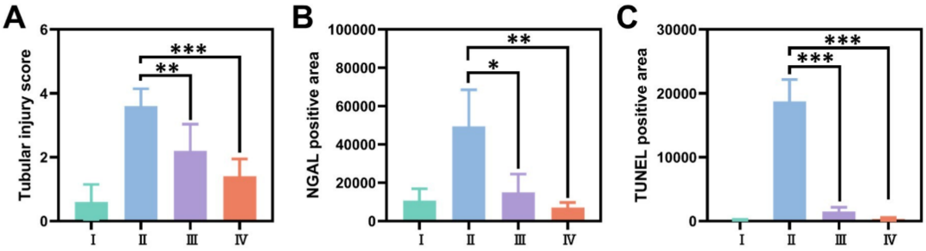

Figure S8. (A) Tubular injury scores quantified from PAS-stained sections. (B) Quantification of neutrophil gelatinase-associated lipocalin (NGAL)-positive areas from immunohistochemistry. (C) The quantitative analysis of TUNEL staining. Data are mean ± SEM; **p* < 0.05, ***p* < 0.01, ****p* < 0.001 vs. RIRI group. I: Sham; II: RIRI; III: RIRI + PN-CDs1; IV: RIRI + PN-CDs2.

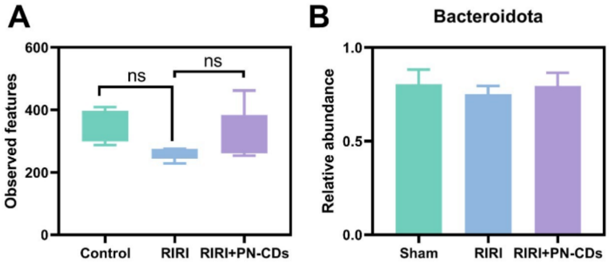

Figure S9. (A) Observed features. (B) Relative abundance of the *Bacteroidota* phylum. ns= no significant.

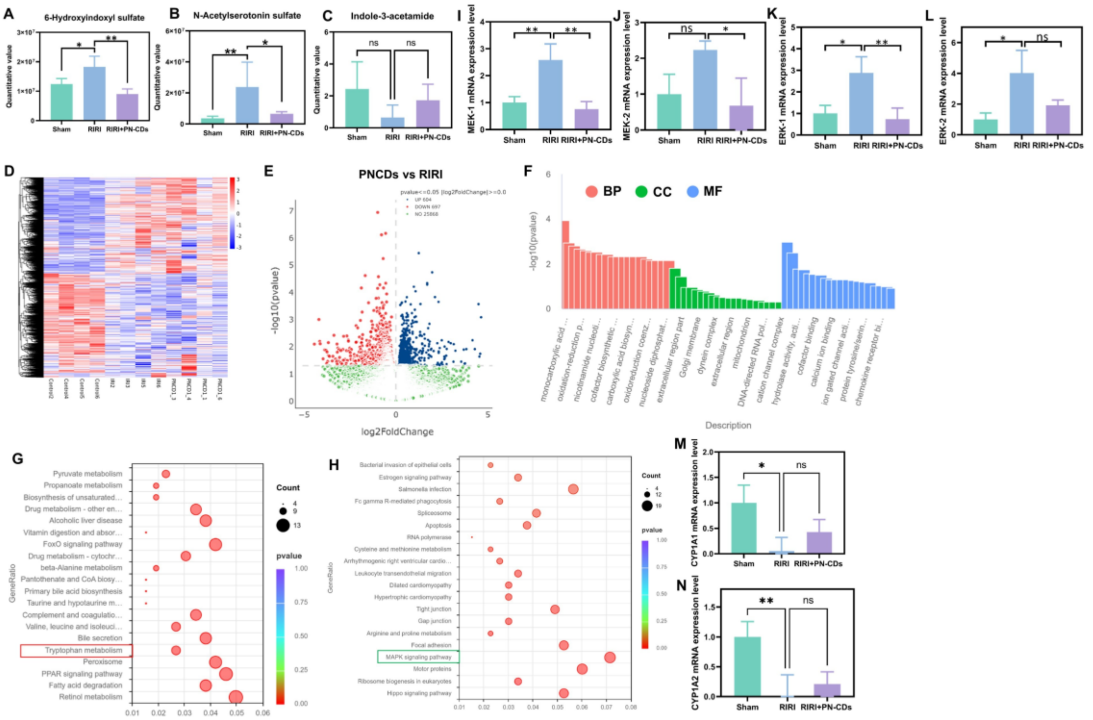

Figure S10. Multi-omics investigation into the mechanism by which PN-CDs alleviate renal injury. (A) Quantification of 6-hydroxyindoxyl sulfate in fecal samples from Sham, RIRI, and PN-CDs-treated mice. (B) Quantification of N-acetylserotonin sulfate in fecal samples. (C) Quantification of indole-3-acetamide in fecal samples. (D) Hierarchical clustering heatmap of differentially expressed genes (DEGs) between the PN-CDs and RIRI groups, revealing distinct transcriptional profiles. (E) Volcano plot of DEGs comparing PN-CDs vs. RIRI, where red and blue dots indicate significantly upregulated and downregulated genes, respectively (**P* < 0.05, $|\log_2 FC| > 0.5$). (F) Gene Ontology (GO) enrichment analysis of DEGs, categorized into biological process (BP, red), cellular component (CC, green), and molecular function (MF, blue). (G) Bubble plot of KEGG pathway enrichment for downregulated DEGs. (H) Bubble plot of KEGG pathway enrichment for upregulated DEGs. qPCR analysis of MEK-1 (I) MEK-2 (J) ERK-1 (K) ERK-2 (L) CYP1A1 (M) CYP1A2 (N) mRNA expression in renal tissues. Data are mean ± SEM; ns, not significant, **P* < 0.05, ***P* < 0.01, ****P* < 0.001 vs. RIRI group.

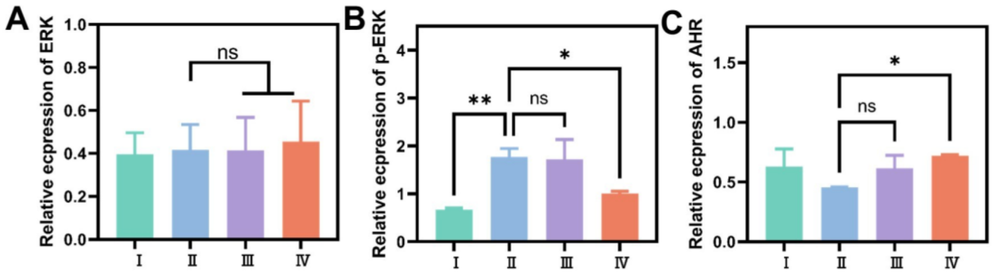

Figure S11. Statistical analysis of Western blot results. (A) Quantification of ERK protein expression; (B) phosphorylated ERK (p-ERK) protein expression; (C) AHR protein expression. Data are presented as mean ± SD. Statistical significance was determined by ANOVA followed by Tukey's post-hoc test (n = 3). ns, not significant, **P* < 0.05, ***P* < 0.01.I: Sham; II: RIRI; III: RIRI + PN-CDs1; IV: RIRI + PN-CDs2.
